# Combining mechanistic quantitative systems pharmacology modeling and patient-derived organoid testing in MET-aberrant non-small cell lung cancer for high-throughput combination efficacy analysis and personalized treatment design

**DOI:** 10.3389/fphar.2025.1685468

**Published:** 2025-11-24

**Authors:** Jingjing Hu, Yanyong Zhao, Qi Rao, Geli Li, Zichen Jiao, Haoxiang Wang, Yuchen Qu, Shihui Xu, Zhongze Gu, Tao Wang, Zaozao Chen, Chen Zhao, Guohua Zhou

**Affiliations:** 1 Department of Clinical Pharmacy, Jinling Hospital, Affiliated Hospital of Medical School, Nanjing University, Nanjing, China; 2 School of Pharmacy, Nanjing Medical University, Nanjing, China; 3 Department of Thoracic Surgery, Nanjing Drum Tower Hospital, Affiliated Hospital of Medical School, Nanjing University, Nanjing, China; 4 Jiangsu Avatarget Biotechnology Co., Ltd., Nanjing, China; 5 State Key Laboratory of Digital Medical Engineering, School of Biological Science and Medical Engineering, Southeast University, Nanjing, China

**Keywords:** MET, quantitative systems pharmacology, virtual clinical trials, combination therapy, patient-derived organoid

## Abstract

Non-small cell lung cancer (NSCLC) harboring MET exon 14 skipping mutations or MET overexpression/amplification typically exhibits highly proliferative and invasive phenotypes and is a significant threat to human health. Although tyrosine kinase inhibitors targeting MET have been approved for clinical use over the past decade, treatment for MET-aberrant patients still face large unmet needs with issues such as limited response duration and drug resistance, low response rates and need for effective combination therapies, differential treatment response in patient subgroups, as well as clinical dose optimization and possibility of personalized medicine. To address these challenges, we developed a quantitative systems pharmacology (QSP) model that mechanistically recapitulated the complex regulation within the MET signaling network and integrated multiscale preclinical-clinical datasets for a total of 16 candidate drugs to drive translational drug research. This comprehensive QSP model framework, upon rigorous stepwise calibration and validation, has enabled high-throughput clinical efficacy analysis of different emerging combination therapies across varying dose ranges, offering crucial insights for drug development and dose optimization in MET-aberrant patients. We further integrated cancer patient-derived organoid (PDO) data on drug sensitivity into the QSP framework and explored the translational utility of this hybrid drug analysis paradigm towards the design of optimal personalized treatment regimens for 5 NSCLC patients harboring MET amplification. To our knowledge, our work is the first multiscale QSP investigation of MET dysregulation for translational cancer drug research, and by integrating QSP model analyses with PDO data it has opened up a new route to facilitate future cancer personalized medicine.

## Introduction

c-MET (or MET), a receptor tyrosine kinase (RTK), is expressed by various epithelial cells in human, including those of the lung, liver, kidney, pancreas, and prostate. It plays a crucial role not only in tissue repair and regeneration, but also in tumor cell survival, proliferation, and metastasis through its various downstream pathways (Kim and Salgia, 2009; Fu et al., 2021). The MET protein is comprised of several regions: the extracellular region containing the SEMA domain (critical for ligand binding, specifically hepatocyte growth factor–HGF) and the PSI domain, the transmembrane region, and the intracellular region containing the juxtamembrane (JM) domain and the tyrosine kinase (TK) domain. On one hand, phosphorylation of Y1349 and Y1356 residues within the TK domain creates a multifunctional docking site and can activate downstream PI3K/AKT and MAPK (mitogen-activated protein kinase) signaling pathways. On the other hand, phosphorylation of Y1003 within the JM domain can facilitate the recruitment of the E3 ubiquitin ligase Cbl, which mediates MET ubiquitination and subsequent degradation thus negatively regulating MET signaling (Uchikawa et al., 2021). Under physiological conditions, these pathways are precisely controlled to regulate cell proliferation and survival. However, MET amplification (a common cause for MET overexpression) would result in increased MET autophosphorylation in cells, and this will lead to overactivation of the downstream pathways to promote cancerous cell proliferation (Tanizaki et al., 2011a). In cells with MET exon 14 skipping (*MET*ex14) mutations, normal function of Y1003 residue (contained in exon 14) is altered or lost, and this impairs Cbl binding to MET and consequently downregulates MET ubiquitination and degradation. As a result, *MET*ex14 can lead to elevated MET protein expression and sustained activation of downstream signaling that ultimately promotes cancerous cell proliferation and invasion (Spitaleri et al., 2023; Cortot et al., 2017; Drilon, 2016). Epidemiological studies indicated that among patients with NSCLC, approximately 3%–4% exhibit MET exon 14 skipping mutations (Socinski et al., 2021). These patients typically do not have other co-occurring mutations. Additionally, primary MET amplifications are present in about 5% of NSCLC patients, while 15% of NSCLC patients can have secondary MET amplification (Yang et al., 2023). In addition, in NSCLC, a number of other RTK pathways such as EGFR, ALK, and ROS1 (upon ligand binding or mutations) can also crosstalk and dynamically interact with the MET pathway to collectively regulate cell growth and survival and thus contribute to tumorigenesis (Remon et al., 2021; Fu et al., 2022; Korpanty et al., 2014; D, 2024). Thus, drug research and development are still in full swing in this field in terms of developing new targeted drugs, new therapeutic modalities, as well as new combination strategies.

Over the past decade, various targeted therapies have been developed for the treatment of MET-aberrant NSCLC. Among them, a major class is MET tyrosine kinase inhibitors (TKIs) including capmatinib, tepotinib, crizotinib, and savolitinib (Mazieres et al., 2023a; Mathieu et al., 2022). These TKIs specifically bind to the ATP-binding pocket of MET to inhibit receptor phosphorylation and activation, thereby blocking downstream proliferative signaling and suppressing tumor growth. The primary approved indication for these TKIs is metastatic NSCLC with *METex14* mutations given the several successful large scale randomized control studies. In addition to MET TKIs, other targeted therapies including inhibitors targeting downstream signaling hub proteins such as MEK, ERK, AKT, and PI3K have also emerged in preclinical investigations and translational studies relating to MET-aberrant cancers (Hu et al., 2025; Qin et al., 2023; Turke et al., 2012). A major challenge in the clinical treatment of MET-aberrant NSCLC is the relatively low patient response rate and limited response duration. For example, Tepotinib was reported to achieve an objective response rate (ORR) of 45% and median duration of response (DoR) of 12.6 months in previously treated *MET*ex14 patients in the VISION trial; similarly, in GEOMETRY mono-1 trial, the ORR and mDoR readouts for Capmatinib are 41% and 9.7 months respectively (Wolf et al., 2020; Mazieres et al., 2023b). Secondly, compared to the *MET*ex14 patient subgroup, only a limited number of clinical investigations were conducted in MET-overexpressed/amplified NSCLC patients, so there is an obvious lack of clinically-relevant evidence to drive translational research. In addition, individual NSCLC MET-aberrant patients can usually exhibit highly distinct response profiles to the same treatment. Thus, how can *in vitro* testing data using patient-derived tumor tissues be utilized to infer clinical response and thereby design optimal treatment strategy for individual patients is always a challenging task with high unmet need (Rivera-Concepcion et al., 2022; Meyer et al., 2024). Consequently, drug development efforts are still active in this field to deliver more effective drugs and new treatment combinations for patients. Novel approaches such as computational modeling will be indispensable towards that goal given that real world clinical trials are exceptionally costly and time-consuming.

Given the complexity of MET signaling in regulating NSCLC tumorigenesis and the long-standing high costs of moving new therapies along the clinical trial journey, quantitative systems pharmacology (QSP) modeling can be very useful in this scenario. As a new paradigm in modern model-informed drug development, QSP can serve as a powerful methodology to quantitatively predict drug efficacy by integrating multilevel mechanisms and multimodal data, thereby facilitating drug discovery and the design of new clinical treatment strategies. In the field of NSCLC drug research (particularly for immuno-oncology drug candidates), QSP modeling has been increasingly explored during translational drug development stages to facilitate clinical trial design (Wang et al., 2023; Schirru et al., 2024). For the investigation of MET-related therapeutics, however, only Jafarnejad et al. have developed a systems biology-type model that described HGF/MET signaling in cancer and simulated different pathway interventions at the *in vitro* level (Jafarnejad et al., 2019). There were no QSP modeling attempts yet for MET-centered drug development purposes that can faithfully connect multiscale preclinical data to population-level clinical efficacy predictions to uncover new combination approaches and advance personalized medicine. Therefore, we have here developed a first-of-its-kind QSP model focusing on MET-aberrant NSCLC that has incorporated detailed mechanistic features from the complex cellular signaling level all the way to the heterogeneous patient level. This QSP model was rigorously calibrated and validated by referencing extensive experimental datasets (including data on signal transduction, cell viability, pharmacokinetics, tumor growth inhibition and clinical-level treatment response) for over 15 different candidate drugs for MET-aberrant NSCLC. Using this model, we analyzed potential population-level treatment efficacy of different therapeutic regimens and combinations, and we found that significantly enhanced patient response can be achieved by using MET TKI together with small molecule inhibitors targeting downstream signaling hub proteins such as PI3K/AKT. In addition, we demonstrated the unique potential of this QSP framework in guiding clinical dose optimization for MET TKIs in NSCLC. We further explored the translational utility of our model-based paradigm employing both high-throughput simulations as well as cancer patient-derived organoid data in guiding individualized treatment design. Our work is the first multiscale QSP modeling effort for the translational drug development in MET-aberrant NSCLC and it can serve as a unique data-driven disease modeling platform for future personalized medicine research in cancer.

## Materials and methods

### Model formulation, calibration and validation using multiscale data

The overall QSP model is constructed based ordinary differential equations (ODEs) derived from mass action kinetics and Hill-type reaction laws (including approximately 130 molecular species and 69 equations, including pharmacokinetic modules of 16 drugs). Intracellular signal transduction reactions under the EGFR, MET, ALK and ROS1 pathways (e.g., receptor-ligand binding, species degradation, phosphorylation, and downstream kinase activation) are primarily described by mass action kinetics, and the therapeutic mechanisms of action of certain cytotoxic drugs are modeled using Hill functions. The model incorporated seven tyrosine kinase inhibitors (TKIs): crizotinib, tepotinib, capmatinib, savolitinib, cabozantinib, erlotinib, and gefitinib, as well as small molecule inhibitors targeting four other kinases: PI3K, AKT, MEK, and ERK. Five chemotherapeutic agents, including cisplatin, carboplatin, pemetrexed, docetaxel, and gemcitabine, are also included. The PK profiles of all drugs included were described using standard ODE-based one or two compartment models, and drug exposure within the tumor was assumed to be proportional to drug exposure in the plasma by using calibrated partition coefficients.

During model calibration, we first utilized cell-level experimental data (cell line experiments and patient-derived organoids) including measurements of signal transduction and drug-induced cell viability change. We used A549 (a non-small cell lung cancer cell line with normal EGFR and MET) as the reference cancer cell line, and based on quantitative literature data, the number of MET and EGFR receptors per A549 cell was determined to be 13,590 and 158,834, respectively (Wisniewski et al., 2014). Considering that MET and mutated ALK and ROS1 can induce spontaneous activation even in the absence of ligands, we allow all model species to reach their respective steady states which were then used as initial conditions when simulating other perturbations and drug treatment. Since MET proteins with exon 14 skipping mutation lack the ubiquitination enzyme binding site and are deficient in ubiquitin-mediated degradation, the degradation rate of MET with exon 14 skipping mutation was set to be lower than that of wild-type MET. ImageJ software (NIH, Bethesda, USA) was used for densitometric quantification of published Western blotting data, and the resulting experimental data values were normalized to their respective maximum or their respective control values (means for such values were used). For cell viability fittings, the resulting cell growth under drug treatment were normalized to the cell growth under untreated condition over the same period of time to obtain the simulated cell viability curves. All model reactions were implemented in MATLAB Simbiology Toolbox (MathWorks, Natick, MA, USA) and simulated using the ode15s solver. Parameter values were either determined based on experimental data (e.g., association/dissociation rates, protein half-life) or through data fitting. The patternsearch function in the Global Optimization Toolbox of MATLAB was used for parameter estimation and optimization. For detailed description of model mechanisms and reactions, please refer to Supplementary Table S1. For model calibration at the *in vivo* level, we reset the tumor doubling time by fitting against experimental tumor growth under the control (no treatment) condition. Then, mouse PK were added to connect drug dosing to drug-mediated tumor growth inhibition *in vivo*; for drugs with only human PK data but no mouse PK data available, reverse allometric scaling was used to derive model parameters for mouse PK. Although such reverse scaling cannot take into account potential species-specific differences in drug metabolism and may lead to inaccuracy in mouse PK extrapolation, we think it will not influence overall model utility at the clinical level as such procedures were only carried out for 3 drugs at the preclinical level.

When formulating the virtual patients and virtual patient population, it was assumed that tumors grow in spherical shapes and its diameter changes (in percentages, compared to baseline) were used for response assessment using the RECIST v1.1 criteria, similar to the approaches used in (Sové et al., 2020; Wang H. et al., 2022; Yang et al., 2025). To account for physiological interindividual variability at the patient level, we allowed key model parameters such as the activation rates of proteins MET, AKT, and ERK as well as cell growth and death rates to vary within reasonable numerical ranges when transitioning from preclinical to clinical. For virtual patients with *METex14* mutation, their tumors were set to contain both MET exon 14 skipping cells and normal cancer cells; for virtual patients with MET amplification, their tumors were set to contain both MET-amplified cells and normal cancer cells. We allowed growth rates of different cell populations to vary among different model-generated individual virtual patients, so that different cancer cells would together grow to a certain tumor volume (threshold), at which we set that as the initial tumor burden for that individual patient and would then begin clinical-level drug treatment simulations. Thus, ratios of different cancer cell populations were varying from patient to patient in our simulations and are dynamically changing overtime. When running virtual clinical trials against real clinical data, we conducted 100 repeated runs for all scenarios to obtain the response interval of the virtual patient population (by recording each individual’s best response in waterfall plots). The percentages of patients achieving complete response (CR) and partial response (PR) were summed to get the objective response rate (ORR) readouts in each of the 100 runs, and the Kolmogorov-Smirnov test was used to assess whether the 100 sets of response readouts conform to a normal distribution. Then, 95% prediction interval of the virtual patient population’s ORR was calculated for each drug dosing scenario.

### Collection of clinical tumor samples

The lung cancer samples used in this study were obtained from Nanjing Drum Tower Hospital. After aseptic cleaning, a small amount of postoperative tumor tissue samples from patients were soaked in tissue preservation solution (AVATARGET, AVA-TPS-0001) and promptly transported to the laboratory for processing and analysis. The diagnosis of these lung cancer samples was confirmed through pathological and imaging examinations. Following approval by the ethics committee (ethics approval file number 2024-206–02) and obtaining informed consent from the patients, the tumor tissue samples were used for lung cancer organoid culture and original tumor characteristic analysis.

### Primary organoid culture

The lung cancer tissues were transported to the laboratory within 6 h post-surgery for processing and primary cell extraction. The samples were initially washed three times with PBS containing 1% penicillin-streptomycin and then cut into approximately 1 mm^3^ fragments using sterile ophthalmic scissors. Tissue digestion was conducted at 37 °C for 15–30 min using an appropriate amount of AVATARGET primary organoid digestion solution (AVA-TPTS-0001). The digestion progress was observed under a microscope every 5 min until the fragments were digested into large cell clusters, each containing 3-5 cells. The digestion process was halted by adding three times the volume of PBS. The filtrate, after passing through a 100 μm filter (BD Falcon, 352360), was centrifuged at 4 °C and 400 g for 5 min. If the red blood cell content was high, red blood cell lysis buffer (Solarbio, SL1070) was added and incubated for about 2 min. After centrifugation, the collected cell pellet is resuspended with an appropriate amount of Matrigel (Corning, 356231) and seeded into a 24-well plate (Corning, 3524) at a volume of 25 μL per well. After a 30-min incubation at 37 °C, each well is supplemented with 500 μL of lung cancer organoid culture medium (AVATARGET, AVA-RTLD-0001) for cultivation under conditions of 37 °C and 5% CO_2_.

### Organoid passaging culture and mutation gene analysis

According to the growth status of organoids, the culture medium is changed every 2–4 days, and the organoids are passaged approximately every 7 days. Generally, each well of organoids is passaged and expanded in a ratio of 1:2-4. Pre-cooled PBS is used to collect the Matrigel and organoid mixture from the well plate, followed by digestion with organoid passaging digestion solution (AVATARGET, AVA-TPOD-0001) for about 5–20 min. The dissociated organoid suspension is collected and mixed evenly with Matrigel, and then reseeded in a volume of 25 μL per well in a 24-well plate. After gelation at 37 °C for 30 min, 500 μL of fresh lung cancer culture medium is added to each well. The successfully constructed lung cancer organoids are passaged to the P3 generation, and subjected to whole exome sequencing (WES) along with the original tumor tissue to analyze and identify samples containing MET gene mutations for subsequent *in vitro* drug sensitivity experiments.

### Drug sensitivity testing

To conduct sensitivity testing with different drugs, certain amounts of organoids were collected and digested to a size of approximately 30μm, then suspended in Matrigel and seeded in a 96-well plate (Corning, 3599) at a density of around 3000 cells per well. After allowing the gel to solidify for about 30 min, the lung cancer complete culture medium was added. After approximately 3 days of culture, the organoids grew to a diameter of around 50 μm. Different concentrations (0, 0.01, 0.1, 0.3163, 1, 3.163, 10 μM) of six targeted drugs (erlotinib, sorafenib, regorafenib, afatinib, cabozantinib, donafenib) and four combinations of chemotherapy drugs (gemcitabine + cisplatin, pemetrexed + cisplatin, docetaxel + cisplatin, paclitaxel + cisplatin) were tested on organoids No.62/104/154. Concentration gradients of 0, 0.01, 0.03, 0.1, 0.3, 1, 3, 10 μM were applied to organoid No.108, which included four targeted drugs (erlotinib, gefitinib, lapatinib, crizotinib) and one chemotherapy drug (idarubicin). Organoid No.125 was treated with concentration ranges of 0, 0.1, 0.3163, 1, 3.163, 10, 31.63, 100, 316.3μM, including two targeted drugs (regorafenib, futibatinib) and nine chemotherapy drugs (paclitaxel, fludarabine, oxaliplatin, gemcitabine, pemetrexed, 5-FU, irinotecan, docetaxel, and chlorambucil). All drugs were purchased from MCE. Preparing the required drug working solutions according to the protocol and adding 200 μL per well, three replicates were set for drug treatment, and images were collected on days 0, 4, and 7. After 7 days of drug treatment, cell viability was analyzed using CCK8 assay (Solarbio, CA1210-500) to calculate the IC50 values and compare the sensitivity of organoids to different drugs.

### Global sensitivity analyses

Partial Rank Correlation Coefficient (PRCC) analysis was performed according to the method described by Marino et al. (2008). For the algorithm setup, latin hypercube sampling with 5000 iterations was applied to each input condition, and the parameter value ranges in the PRCC calculations were set to one-third to three times of their respective baseline values. We simulated both preclinical tumor volume changes on day 90 under drug-free conditions and tumor volume changes on day 90 under capmatinib treatment, and parameters significantly correlated with the model output under each condition were presented separately.

## Results

### Overview of QSP model structure and mechanisms

**FIGURE 1:**
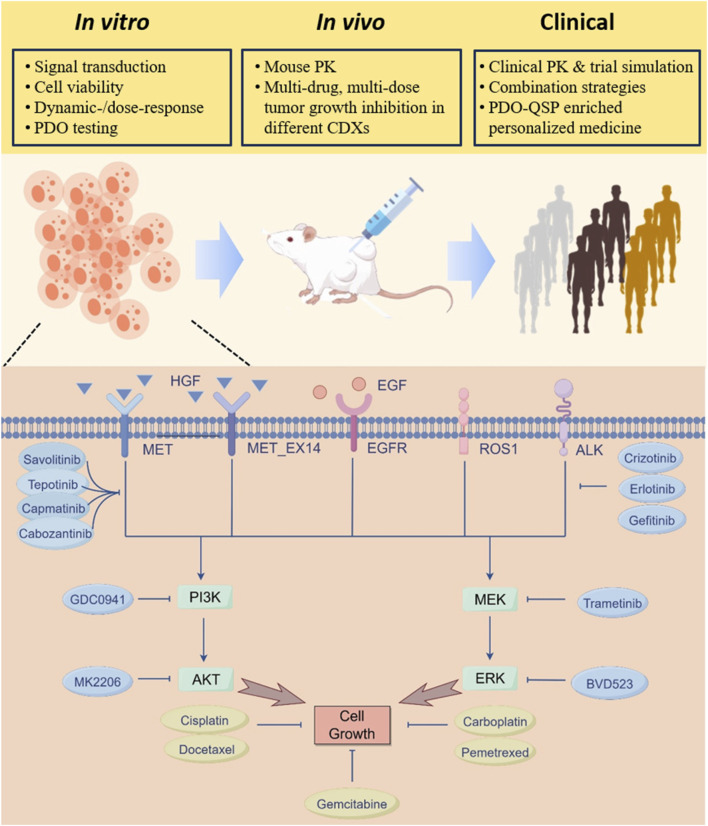
QSP model overview and flow of multiscale model calibration, validation and analyses. A mechanism-based multiscale QSP model was formulated to investigate the therapeutic potential of various intervention strategies in NSCLC patients exhibiting MET aberrancy. At the cell signaling level, key receptor tyrosine kinase pathways implicated in NSCLC cell proliferation including MET, EGFR, ALK, and ROS1 were incorporated and validated against multi-modal *in vitro* data. The cell-level model was then extended to the preclinical *in vivo* level by adding pharmacokinetic components of different drugs, and the model was calibrated against tumor growth profiles under a number of treatment scenarios in mice. Then at the clinical level, the QSP model was updated and utilized to create virtual patient populations that can accurately simulate clinical efficacy readouts of different MET-targeted therapies. Additional clinical level analyses include assessing new combination treatment strategies, evaluating possibility of dose optimization, and predicting individualized treatment plans by incorporating patient-derived organoid testing data.

The full QSP model is comprised of a cell signaling module and multiple drug pharmacokinetics (PK) modules to systematically describe the mechanisms by which MET pathway dysregulation promotes tumor growth and how targeted pathway interventions inhibit tumor growth (Figure 1). The cell signaling module characterizes the pro-tumorigenic intracellular pathways and crosstalks collectively regulated by RTKs including MET, EGFR, ROS1, and ALK. Specifically, binding of HGF to MET induces potent MET phosphorylation and subsequent downstream activation of AKT and MAPK signaling pathways which positively drive cancer cell proliferation. The model also incorporated the ligand-independent auto-activation mechanism of MET, whereby overexpressed MET receptors can autophosphorylate in the absence of HGF to drive the cascaded activation of downstream signaling (Graveel et al., 2013). Furthermore, the model considered the mechanisms of EGFR phosphorylation and signaling activation under epidermal growth factor (EGF) stimulation. It also included the processes by which ALK gene mutations and ROS1 gene rearrangements can lead to their own phosphorylating activation and upregulation of downstream cell growth-related pathways. For drug targeting, in the QSP model we included the mechanisms of action of various candidate drugs spanning three different classes (MET inhibitors, inhibitors of other intracellular signaling kinases, and chemotherapeutic agents), including capmatinib, tepotinib, savolitinib, cabozantinib, crizotinib, erlotinib, gefitinib, GDC0941, trametinib, MK2206, BVD523, pemetrexed, docetaxel, carboplatin, and cisplatin. The differential *in vivo* disposition processes of the drugs were described by compartmental models. Small molecule drugs were assumed to directly act on their corresponding targets to downregulate protein phosphorylation (for MET inhibitors, of note, mechanistic association and dissociation between drugs and receptor targets were modeled), while chemotherapeutic agents were assumed to directly increase the cell death rate in the model. Details of model species, reactions and descriptions are provided in the Supplementary Material.

### Stepwise model calibration and validation using multimodal pathway signaling and cell viability data

**FIGURE 2:**
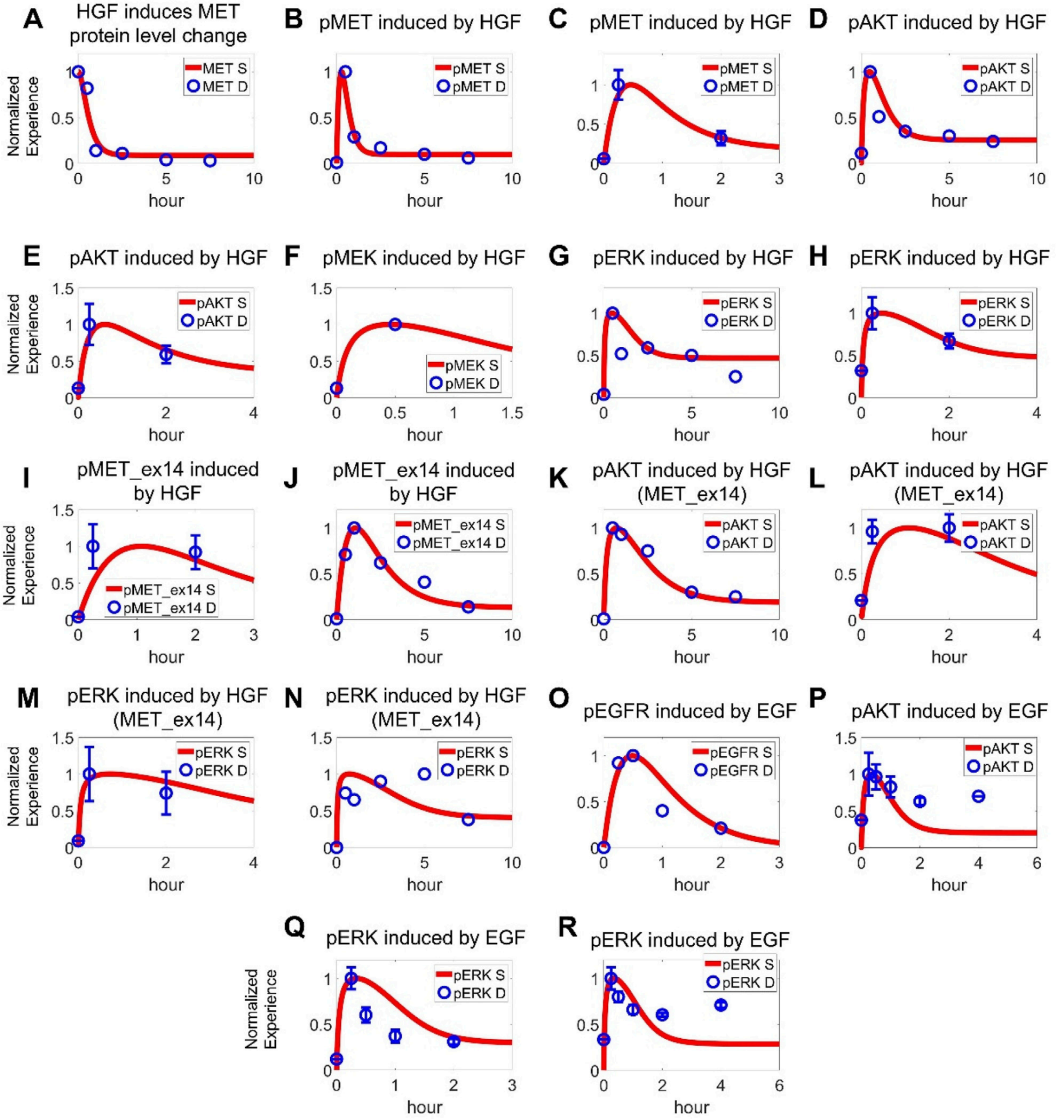
Cell-level QSP model calibration using pathway signal transduction data. In cancer cells with normal MET expression, **(A)** HGF (100 ng/mL) induces internalization and degradation of MET receptors, along with **(B,C)** phosphorylating activation of MET (**B** 100 ng/mL, **C** 50 ng/mL) (Wang F. et al., 2022; Fernandes et al., 2023). **(D,E)** HGF (100 ng/mL, 50 ng/mL) induces downstream activation of AKT (Wang F. et al., 2022; Fernandes et al., 2023). **(F)** HGF (50 ng/mL) induces downstream activation of MEK (Yang et al., 2020). **(G,H)** HGF (100 ng/mL, 50 ng/mL) induces downstream activation of ERK (Wang F. et al., 2022; Fernandes et al., 2023). In cancer cells with MET exon 14 skipping mutations, **(I,J)** HGF (50 ng/mL, 100 ng/mL) induces prolonged phosphorylation of MET as well as **(K,L)** AKT activation and **(M,N)** ERK activation (Wang F. et al., 2022; Fernandes et al., 2023). For the EGFR pathway, **(O)** EGF (5 ng/mL) induces phosphorylation of EGFR and **(P)** downstream AKT kinase (100 ng/mL EGF) (Nishimura et al., 2015; Lee et al., 2011; Shen et al., 2021). **(Q,R)** EGF (5 ng/mL, 100 ng/mL) also induces activation of ERK (Nishimura et al., 2015; Lee et al., 2011). **(A–R)** Y axes are relative expression levels (normalized to their respective maximum values). S, simulation; D, experimental *in vitro* data.

The cell signaling module in our QSP model was primarily calibrated/validated using data obtained from NSCLC cell lines harboring *MET*ex14 mutations or MET overexpression, including A549, H596, and EBC1 cells. Prior to any ligand stimulation or drug administration, all protein levels in the cell signaling module were initialized to the steady states. Then, different experimental interventions were input into the model and simulated outputs were directly compared with the corresponding reported *in vitro* data obtained under the same conditions. To this end, a substantial amount of experimental data was utilized, encompassing the ligand-dependent (e.g., EGF, HGF) activation and drug-induced inhibition of intracellular signaling as well as drug-induced regulation of cancer cell viability (covering both time-dependent and dose-dependent features with over 10 drugs). More specifically, ∼70 sets (with over 450 datapoints) of *in vitro* experimental data for a total of 16 drugs, including seven TKIs (crizotinib, capmatinib, tepotinib, savolitinib, cabozantinib, erlotinib, and gefitinib), four small molecule inhibitors targeting downstream proteins (PI3K inhibitor GDC0941, AKT inhibitor MK2206, MEK inhibitor trametinib, and ERK inhibitor BVD523), and five chemotherapeutic agents (carboplatin, cisplatin, docetaxel, pemetrexed, and gemcitabine) were simultaneously incorporated during model optimization. In a stepwise manner, the model was first optimized using experimental data of HGF-mediated activation of normal MET pathway, including changes in total MET proteins (Figure 2A), phosphorylation level of MET (Figures 2B,C; Supplementary Figures S1A,B), and activation of downstream AKT, MEK and ERK proteins (Figures 2D–H) (Wang F. et al., 2022; Fernandes et al., 2023; Yang et al., 2020). Subsequently, we added mechanisms of *MET*ex14 mutations and quantitatively reproduced the prolonged MET phosphorylation profiles (Figures 2I,J) and data on downstream AKT and MEK/ERK phosphorylation under different concentrations of HGF stimulation as observed in *MET*ex14 cells (Figures 2K–N) (Wang F. et al., 2022; Fernandes et al., 2023; Ahn et al., 2019). Additionally, we also incorporated data related to EGF-induced EGFR phosphorylation (Figure 2P; Supplementary Figure S1C) and downstream activation of AKT and ERK in NSCLC cells (Figures 2O–R) (Nishimura et al., 2015; Lee et al., 2011; Shen et al., 2021). Although minor discrepancies were noted in certain node fittings, the model captures the overall signaling network quite well and we believe that more data would be helpful to further advance the overall model performance.

**FIGURE 3:**
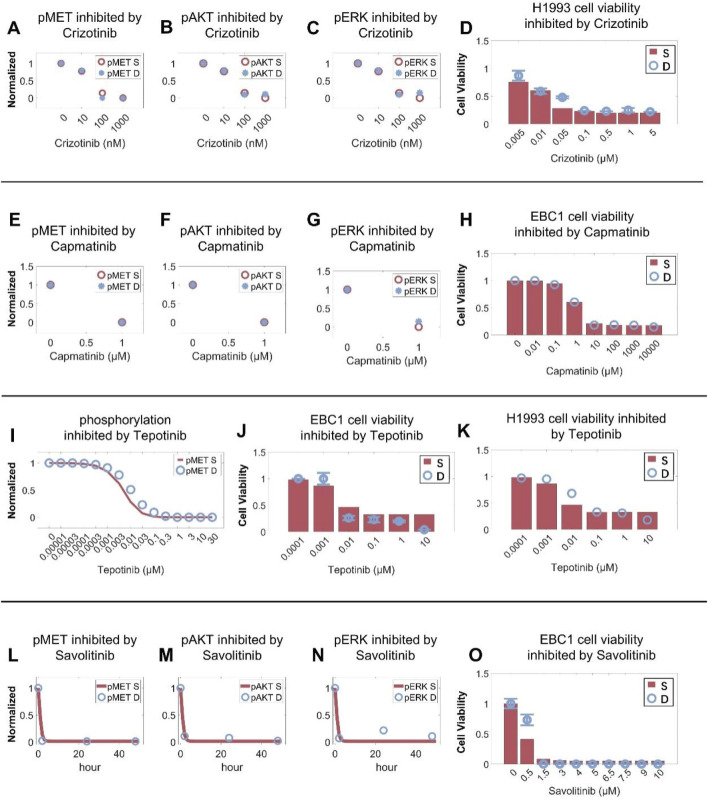
Cell-level QSP model calibration using drug intervention and cell viability data. In the H1993 cell line (MET amplification), crizotinib induces dose-dependent inhibition of **(A)** MET phosphorylation, inhibition of **(B)** AKT and **(C)** ERK activation, and **(D)** cell viability. In the EBC1 cell line (MET amplification), capmatinib induces dose-dependent inhibition of **(E)** MET phosphorylation, inhibition of **(F)** AKT and **(G)** ERK activation, and **(H)** cell viability. In MET-amplified cancer cells, tepotinib inhibits **(I)** MET phosphorylation and **(J,K)** cell viability in a dose-dependent manner. In the EBC1 cell line, savolitinib induces time-dependent inhibition of **(L)** MET phosphorylation, **(M)** AKT and **(N)** ERK activation, and **(O)** cell viability in a dose-dependent manner. **(A–O)** Y axes are relative expression levels (normalized to their respective maximum values). S, simulation; D, experimental *in vitro* data.

Next, we optimized the model by calibrating the inhibitory effects of various targeted inhibitors at different concentrations on intracellular signaling as well as cell viability in NSCLC cell lines. The dose-response profiles of crizotinib, a TKI targeting MET, ROS1 and ALK, were quantitatively reproduced in different cell lines (MET-overexpressing EBC1, ROS1-rearranged HCC78, and ALK-mutant H3122), with increasing crizotinib downregulating the phosphorylation levels of MET, AKT, and ERK as well as leading to reduced cell viability (Figures 3A–D; Supplementary Figures S2A–F) (Calles et al., 2015; Katayama et al., 2011; Yasuda et al., 2012). Capmatinib, tepotinib, and savolitinib are selective MET inhibitors that can specifically bind to MET and inhibit its activation. The cell signaling module successfully simulated the regulatory impact of capmatinib and tepotinib (at varying concentrations) on MET, AKT, and ERK and their inhibitory effects on cell viability in MET-amplified EBC1 and H1993 cells (Figures 3E–K; Supplementary Figures S1D,E) (Kim et al., 2019; Albers et al., 2023; Thu et al., 2024; Berges et al., 2023). Similarly, we also calibrated the model against data describing time-dependent inhibitory effect of savolitinib on MET, AKT, and ERK activation and its collective impact on cell viability (Figures 3L–O) (Henry et al., 2016). Additionally, we utilized experimental data relating to the dose-response inhibitory effect of several other drugs such as cabozantinib (Supplementary Figures S2G,H), MK2206 and BVD523 on cell viability during model calibration (Supplementary Figures S2I–K) (Dai et al., 2013; Karmacharya et al., 2021; Shin et al., 2018). Also, model-based cellular cytotoxicity response to chemotherapeutic agents including carboplatin, cisplatin, docetaxel, pemetrexed, and gemcitabine were also calibrated against *in vitro* data (Supplementary Figures S2L–O) (Shi et al., 2016; Wu et al., 2016; Liu et al., 2017; Kwon et al., 2019).

**FIGURE 4:**
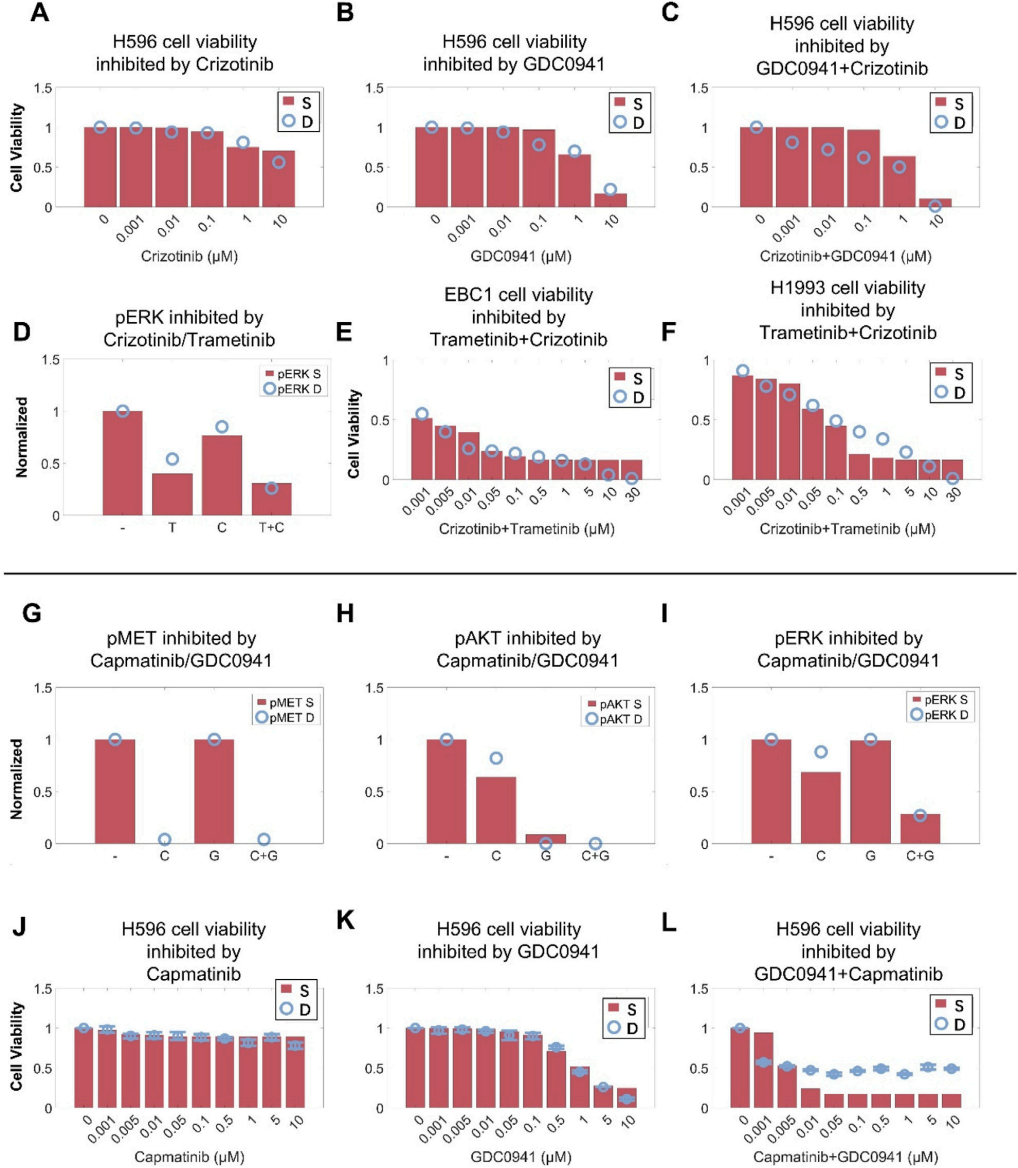
Cell-level QSP model calibration and validation using combination treatment data in different MET-aberrant cell lines. In the H596 cell line (MET exon 14 skipping mutation), the QSP model accurately characterized the cell viability inhibition by **(A)** crizotinib, **(B)** PI3K inhibitor (GDC0941), **(C)** and crizotinib plus GDC0941 combination. In MET-amplified cancer cells, MEK inhibitor (trametinib, denoted as T) and crizotinib (denoted as C) can inhibit activation of **(D)** ERK and **(E,F)** cell viability in a dose-dependent manner. In the H596 cell line, capmatinib or GDC0941 alone or in combination can inhibit **(G)** MET phosphorylation, **(H)** AKT and **(I)** ERK activation, and **(J–L)** cell viability in a dose-dependent manner. **(A–L)** Y axes are relative expression levels (normalized to their respective maximum values). S, simulation; D, experimental *in vitro* data.

To validate the predictive capability of the cell signaling module in our QSP model, we first simulated the inhibitory effects of crizotinib in combination with the PI3K inhibitor GDC0941 on MET pathway signaling and cell viability in the H596 cell line with *MET*ex14 mutations (Figures 4A–C). We also simulated the effect of crizotinib in combination with the MEK inhibitor trametinib on ERK phosphorylation and cell viability in MET-amplified H1993 and EBC1 cell lines (Figures 4A–F). By calibrating only the respective single-agent effect of GDC0941 (on PI3K inhibition) and trametinib (on MEK inhibition) in this scenario, the resulting simulations of such targeted combinations can still match quite well with the experimental data observed in both MET-mutant and MET-amplified cells (Chiba et al., 2016; En et al., 2017). We again validated our model by investigating the capmatinib/GDC0941 combination. Without further changes to the model parameters, the simulations can simultaneously reproduce the complex nonlinear inhibitory effects of capmatinib and GDC0941 (used alone and in combination) on both intracellular signaling (in terms of phosphorylation of MET, AKT and ERK) and cell viability (Figures 4G–L) (Jamme et al., 2020). These results again demonstrated the significant potential of the cell signaling module within our QSP model in predicting dynamic cell signaling changes and drug inhibitory effects under complex targeted intervention conditions.

### Translating the model to *in vivo* level enables quantitative analysis of drug-induced tumor growth inhibition in mice

**FIGURE 5:**
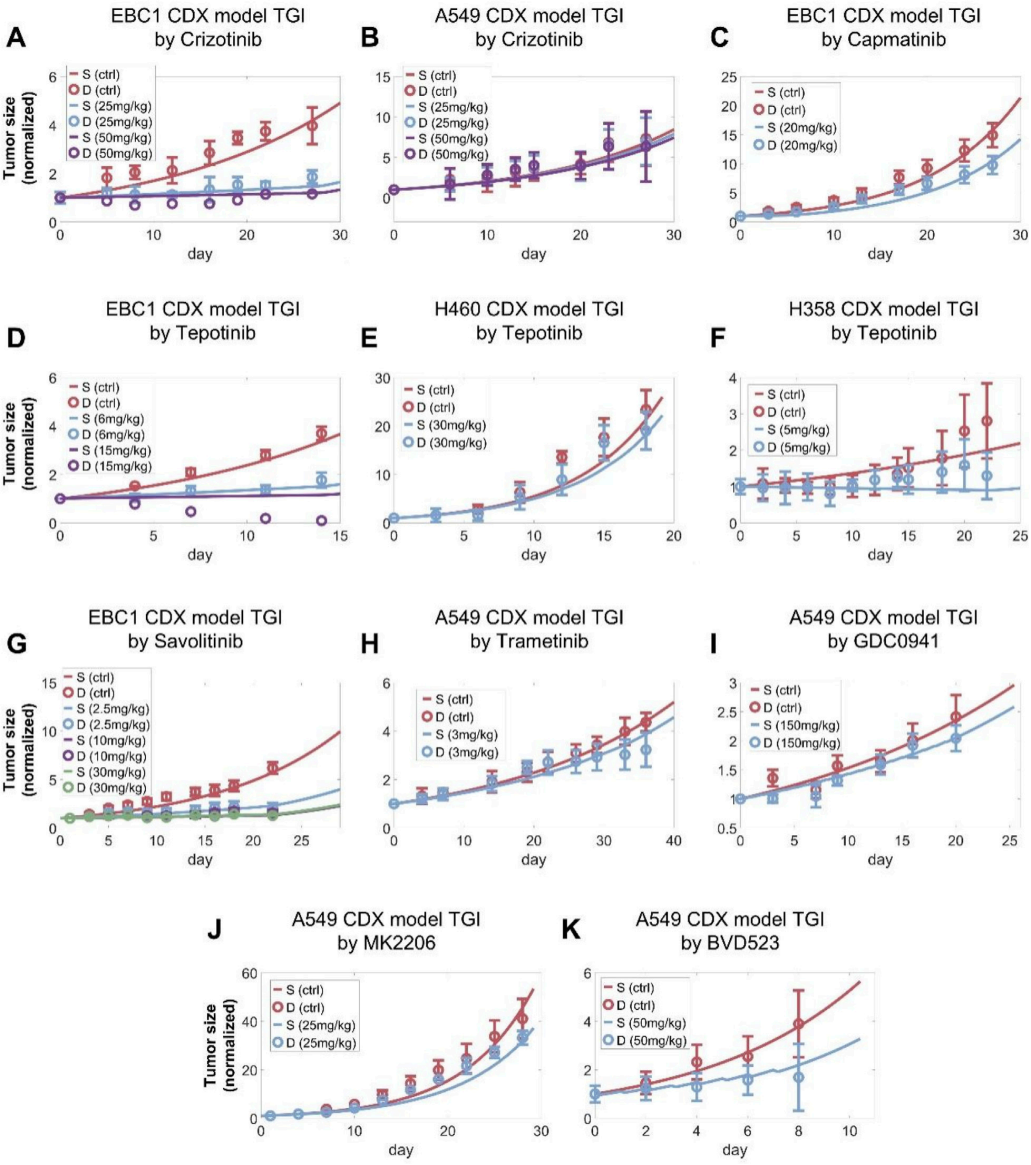
Preclinical *in vivo* QSP model calibration using tumor of tumor size changes in mice model. The QSP model simulations accurately reproduced the reported *in vivo* antitumor activity of **(A,B)** crizotinib, **(C)** capamatinib, **(D–F)** tepotinib, **(G)** savolitinib, **(H)** trametinib (MEK inhibitor), **(I)** GDC0941 (PI3K inhibitor), **(J)** MK2206 (AKT inhibitor), **(K)** BVD523 (ERK inhibitor) at different doses in NSCLC xenograft models. **(A–K)** In the simulations, tumors were allowed to grow to certain volumes before drug administration according to the different studies referenced and the maximum tumor volume was fixed to 2000 mm^3^. All tumor growth profiles were normalized to their starting volumes. The weight of a mouse was assumed to be approximately 20 g to calculate the drug doses administered. S, simulation; D, experimental *in vivo* data.

After the cell signaling module was comprehensively calibrated and validated, we further tested the model’s predictive power at the *in vivo* scenarios. During *in vitro* to *in vivo* translation, we hypothesized that parameters related to intracellular signaling and drug inhibition remained unchanged while parameters related to cell growth and death can vary given the different tissue microenvironment *in vivo*. We also developed corresponding pharmacokinetic (PK) modules for all drugs included in the *in vivo* analysis, through which plasma drug concentration profiles in mice were accurately depicted (Supplementary Figuress S3A–X). After calibrating the tumor growth parameters using the control (no treatment) growth curves observed in the studies, the resulting QSP model can accurately describe all the curated data on drug-induced *in vivo* time-course tumor growth inhibition for a total of 16 drugs and 34 doses tested. For the MET TKIs, our simulations accurately reproduced the experimental observations that these TKIs typically exhibit significantly stronger inhibitory effect on tumors with high MET expression (e.g., EBC1 CDX) compared to those with normal MET (e.g., A549 CDX) (Figures 5A–G) (Henry et al., 2016; Tanizaki et al., 2011b; Ramesh et al., 2023; Wu et al., 2022; Rosell et al., 2024; Bladt et al., 2013). For other targeted kinase inhibitors, as they directly target downstream signaling hubs, they exhibit differential effects in terms of tumor growth inhibition in MET-normal tumors *in vivo* as reproduced by our QSP model (Figures 5H–K) (Sos et al., 2009; Manchado et al., 2016). Similarly, the direct cytotoxic effects of chemotherapeutic agents *in vivo* were also successfully captured by our model simulations (Supplementary Figures S5A–E). These results further demonstrated that our multiscale mechanistic QSP model is an effective computational platform for translational efficacy prediction of potential candidate therapies against MET-aberrant tumors.

### QSP model-based virtual clinical trials enable accurate prediction of clinical patient response to MET TKIs and high-throughput investigation of new dosing regimens

**FIGURE 6:**
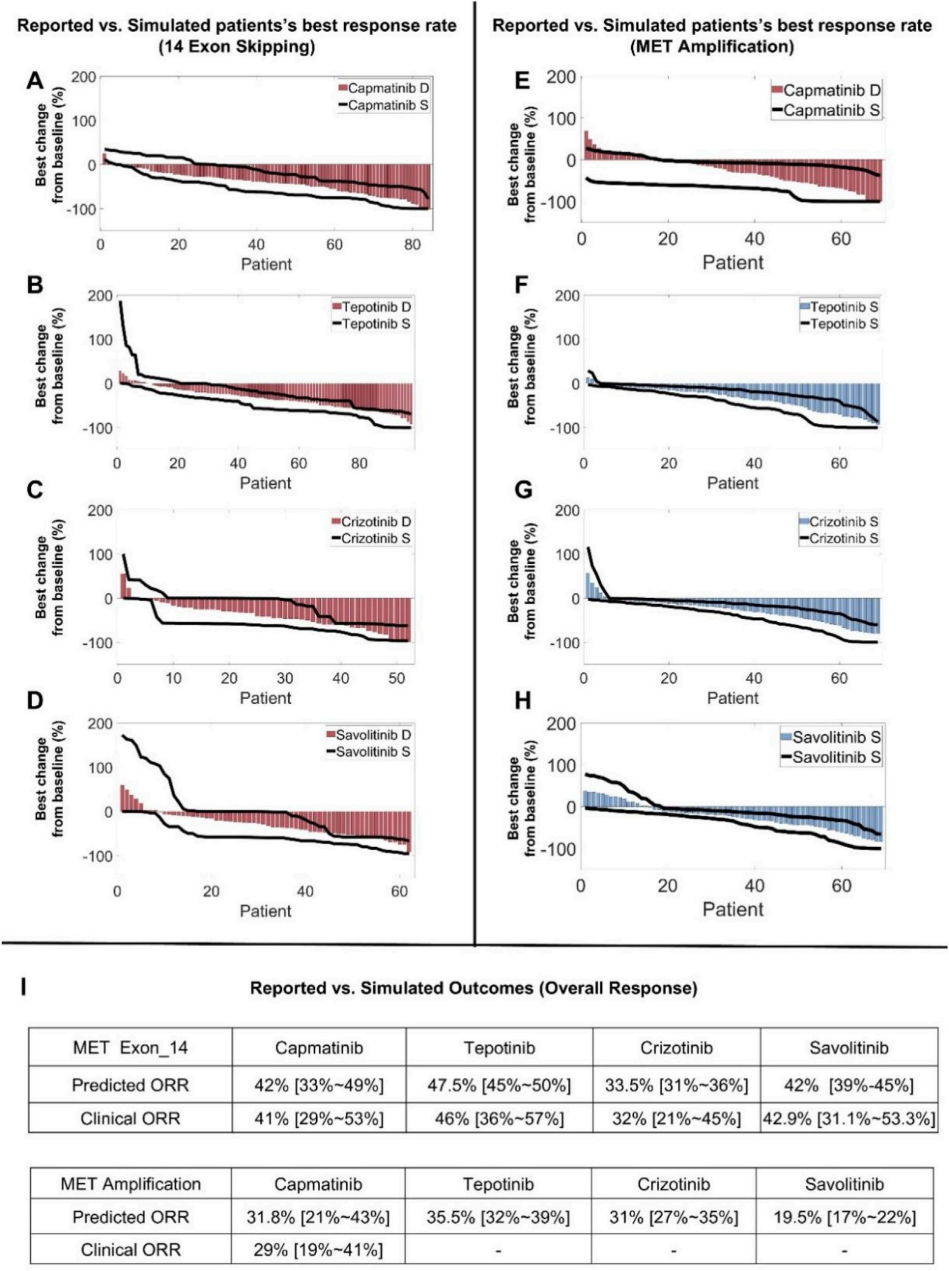
QSP model-based virtual clinical trials enable accurate prediction of clinical patient response to multiple MET TKIs. Using the model-based virtual patient population, **(A–D)** clinical population-level response in terms of best individual tumor change response (in waterfall plots) to different MET TKIs were simulated and compared to reported clinical trial data of NSCLC patients with MET exon 14 skipping mutations. **(E)** Patient response profiles to capmatinib (in waterfall plots) were simulated using the actual clinical dosing schedule and compared with reported clinical trial data in MET-amplified NSCLC patients. **(F–H)** Patient response profiles (MET-amplified NSCLC patients) to another three MET TKIs (tepotinib, crizotinib, and savolitinib) were simulated and displayed in waterfall plots. **(I)** A table that summarizes the predicted ORRs and reported clinical ORRs for different MET TKIs in NSCLC patient populations with MET exon 14 skipping mutations or MET amplification. **(A–E)** The simulated population-level clinical response profiles after drug treatment were quantitatively compared to published clinical trial data, with red bars representing the best percentage change from baseline in tumor size (e.g., sum of lesion diameters) of individual patients as reported in the actual clinical trials and displayed in waterfall plots. The black lines represent the ranges of simulated tumor size changes (from 100 repeated runs) in all individual virtual patients (of the same population size as the actual clinical trial population) displayed in a similar manner. **(F–H)** Blue bars represent the simulated median of the best tumor size change percentage of each individual virtual patient and are displayed in waterfall plots, and black lines represent the ranges of simulated tumor size changes in all individual virtual patients displayed in a similar manner (both from 100 repeated runs). S, simulation; D, clinical trial data.

After the QSP model was rigorously calibrated and validated at the preclinical level, we generated model-based virtual patients to explore clinical level simulations of targeted therapies by starting with the four MET TKIs (capmatinib, tepotinib, crizotinib, and savolitinib). We successfully generated two virtual patient cohorts containing 5000 virtual patients each, representing the NSCLC patients with *MET*ex14 mutations and patients with MET amplification respectively. These patients have successfully gone through a series of screening procedures and they quantitatively differed in their cellular protein expression and activation profiles, tumor growth kinetics, and the proportion of different cancer cell clones within the tumors to together reflect the interindividual variability in MET-aberrant NSCLC patients (see Methods for more details regarding virtual patient generation). Subsequently, we performed virtual clinical trials (for each of the four MET TKIs) according to the exact dosing regimens implemented in the real-world clinical trials for these virtual patients. For NSCLC virtual patients with *MET*ex14 mutations, we sampled the entire population using a calculated prevalence-weighted probability using clinical response data for capmatinib, tepotinib and crizotinib in NSCLC patients as the training set. After 100 sampling repeats of these patients, we found that the respective simulated individual-level and population-level responses for the three MET TKIs can accurately reflect the corresponding reported clinical trial response data (in NCT02414139, NCT02864992, NCT00585195) in terms of both objective response rates and response depth distribution (in waterfall plots) (Figures 6A–C) (Wolf et al., 2020; Paik et al., 2020; Drilon et al., 2020). We then validated the predictive power of this virtual clinical trial approach for another MET TKI, savolitinib, by changing only the drug-specific potency and human PK parameters, and the resulting predicted treatment response can again match the reported clinical data in *MET*ex14 mutated NSCLC patients (Figure 6D) (Lu et al., 2021). We then carried out a similar simulation procedure for NSCLC patients with MET amplification. Our virtual clinical trial approach could accurately capture the varied patient response as observed in the Geometry Mono-1 trial for capmatinib in MET-amplified patients (Figure 6E), and we further predicted the population-level patient response of another three MET TKIs in typical MET-amplified NSCLC patients (Figures 6F–H) (Wolf et al., 2020). The overall results of the above clinical-level simulations with respect to clinical data were summarized in Figure 6I.

**FIGURE 7:**
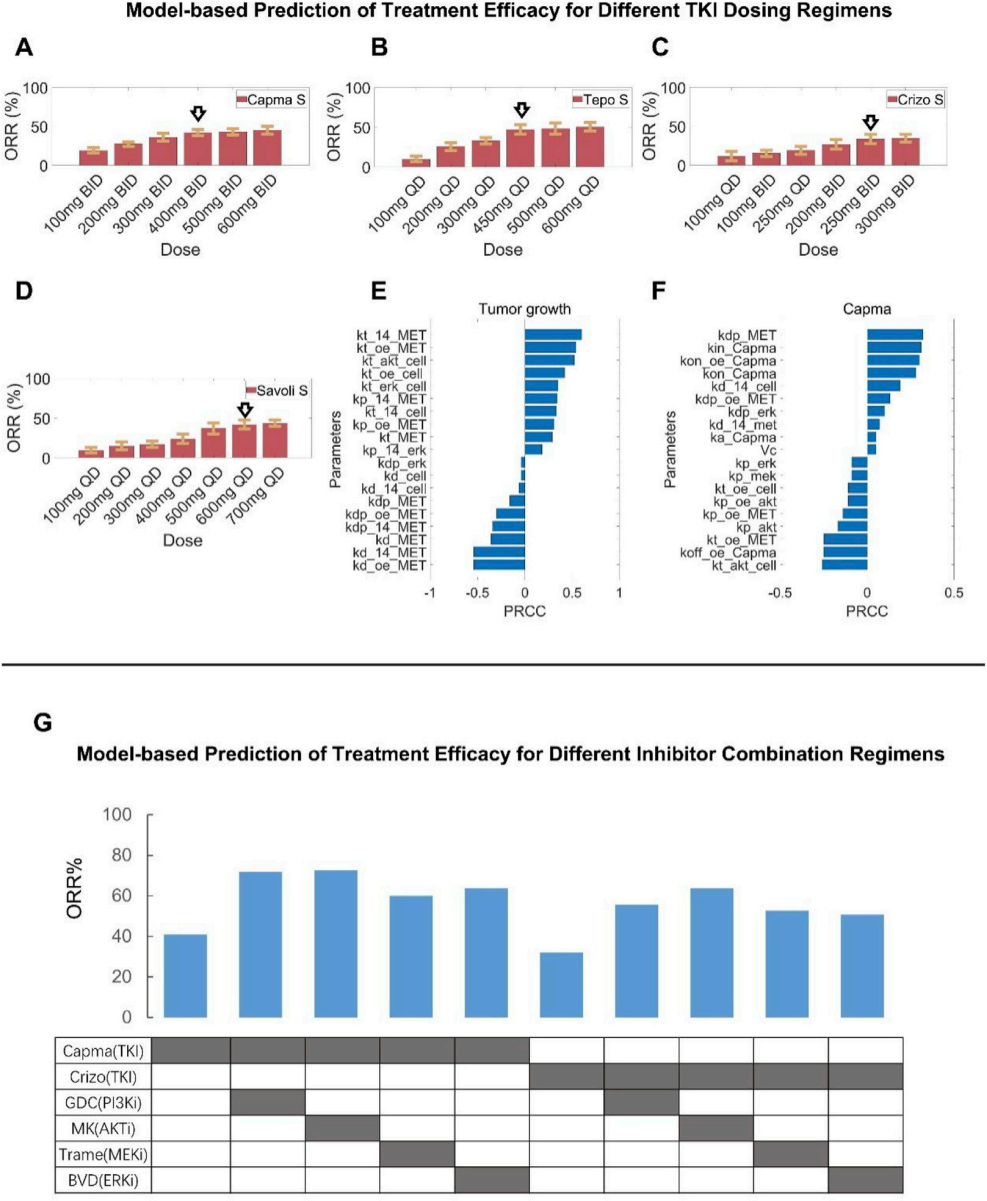
Assessment of alternative clinical dosing regimens and new combination treatment strategies via model-based virtual clinical trials. Using the formulated virtual patient population (NSCLC patients with MET exon 14 skipping mutation), clinical-level response to different MET TKIs at alternative dosing regimens (higher and lower, with the standard clinical dose denoted by arrows) were comparatively simulated, including **(A)** capmatinib, **(B)** tepotinib, **(C)** crizotinib, and **(D)** savolitinib. **(E,F)** To investigate the key parameters influencing tumor volume changes, sensitivity analyses using the partial rank correlation coefficient (PRCC) methodology were performed, including **(E)** sensitive parameters that potently influence tumor volume under the no treatment condition and **(F)** sensitive parameters that potently influence tumor volume change following capmatinib treatment. **(G)** The model-based virtual patient population was utilized to predict the clinical-level response (in terms of ORR) to different new combination regimens, including capmatinib or crizotinib in combination with other protein kinase inhibitors (GDC0941, MK-2206, trametinib, and BVD-523). S, simulation.

To further explore the impact of different treatment regimens for patients with *MET*ex14 mutations, we varied the doses of MET TKIs and simulated the corresponding clinical-level efficacy. The simulation results showed that for capmatinib, the population-level response (in terms of ORR) of the 400 mg BID regimen currently in clinical use would be slightly inferior than that of a higher dose (e.g., 600 mg BID regimen) but superior than that of a lower dose (e.g., 300 mg BID) (Figure 7A). For tepotinib, optimal ORR was predicted to be achieved around the clinically-approved dose of 500 mg QD (contains 450 mg active moiety); even if the dose was increased to 600 mg QD, the simulations showed no increase in patient response (ORR) (Figure 7B). For crizotinib, the predicted ORR of clinical dose (250 mg BID) is very similar to that of alternative regimens (200 mg BID and 300 mg BID) (Figure 7C). For savolitinib, it was predicted that the population-level clinical efficacy would plateau at about 500 mg QD dose, which suggests that clinical dosing of savolitinib (600 mg QD is the approved dose) may be further optimized (Figure 7D).

### Model-based evaluation of new combination treatment strategies involving additional molecular targets within the MET pathway

We conducted global sensitivity analyses using the partial rank correlation coefficient (PRCC) algorithm to assess the influence of model parameters on key outcome measures, namely, tumor volume and the maximum change of tumor diameter at the *in vivo* preclinical level, in different treatment conditions. The analyses results with the top-ranked parameters were summarized in Figures 7E,F. As anticipated, the synthesis and degradation rates of MET (kt_14_MET, kd_oe_MET) can exert significant positive and negative impact on tumor growth. Furthermore, parameters relating to the regulatory potency of protein kinases AKT and ERK on tumor cell proliferation (kt_akt_cell, kt_erk_cell) were also of high impact. In addition, parameters associated with the function of *MET*ex14 mutations (kt_14_MET, kd_14_MET, kp_14_MET, kdp_14_MET, kt_14_cell, kd_14_cell) are influential in driving tumor growth under the control (no treatment) condition. For the sensitivity analysis results under the drug treatment scenario (capmatinib was used as an example) with maximum tumor diameter change as the output of interest, parameters relating to the pharmacological mechanisms of capmatinib such as the rates of capmatinib binding and dissociation (with MET, kon_capma, kon_oe_capma, koff_capma) were significantly parameters in driving tumor regression. Interestingly, parameters relating to the signaling potency of protein kinases (e,g, AKT, ERK) can still also have an impact on tumor regression, albeit at smaller magnitudes compared to the drug-specific parameters.

Based on these findings, we seek to evaluate the translational potential of new combinations for MET-aberrant NSCLC patients by predicting the population-level clinical response to emerging combinations involving MET inhibitors (e.g., capmatinib, crizotinib) and other kinase inhibitors (targeting PI3K, AKT, MEK, and ERK) (Figure 7G). Overall, by integrating the preclinical cell inhibition potency, TGI dynamics, and clinical PK data for each specific kinase inhibitor, our virtual clinical trials predicted that such combinations can generally result in superior patient response (in terms of ORR) compared to monotherapy in *MET*ex14 mutated NSCLC patients. Among which, the combinations of capmatinib with PI3K or AKT inhibitors (using GDC-0941 and MK-2206 as examples) were predicted to achieve the most substantial ORR improvement (by ∼30%) in MET-aberrant NSCLC patients, while the impact of other combinations (e.g., with MEK or ERK inhibitors, using trametinib and BVD-523 as examples) was less pronounced. The resulting clinical efficacy readouts of combinations involving crizotinib were generally lower than the capmatinib-based combinations. Our simulations provided important insights for the development of new combination strategies against MET-aberrant cancers that can potentially improve patient outcome.

### Model-based individualized prediction of optimal clinical treatment regimen using patient-derived organoid testing data

**FIGURE 8:**
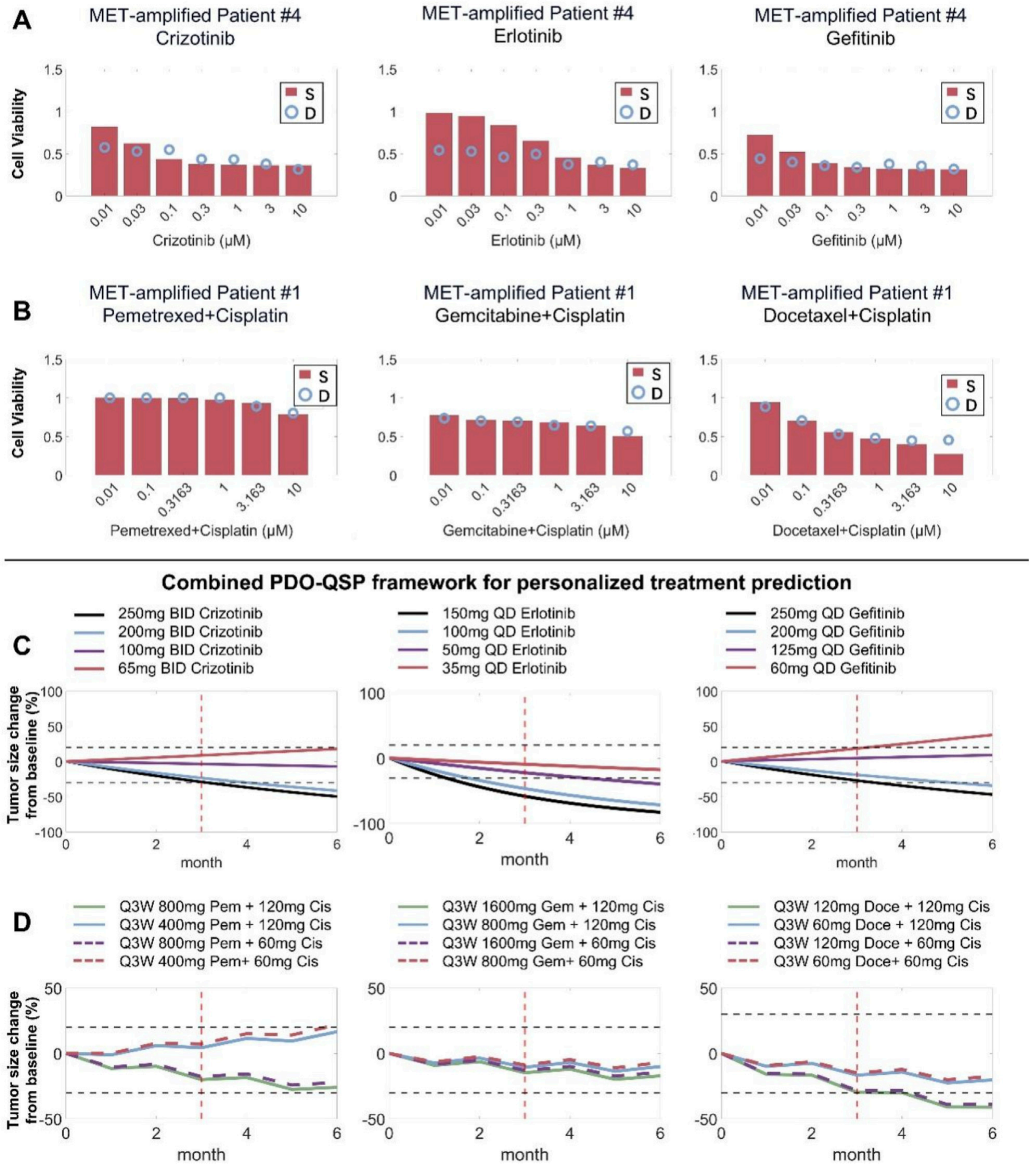
Combining patient-derived organoid testing and mechanistic QSP modeling to project individualized patient treatment response. **(A,B)** Patient-derived organoids (showing patient #4 and #1 here) from MET-amplified NSCLC patients were cultured *in vitro* and treated with different candidate drugs at different doses, and experimental cell viability results were used to inform QSP model parameterization during the formation of individualized virtual patients. **(C,D)** The combined PDO-QSP model framework was then utilized to predict and compare the individual patients’ (#4 and #1) clinical tumor regression profiles under different potential dosing regimens. The horizontal dashed black lines represent thresholds for 20% increase and 30% decrease in tumor diameter, respectively, and the red vertical lines indicate the three-month time point. Standard drug dose/schedule used in simulations: erlotinib – 150 mg QD, gefitinib – 250 mg QD, pemetrexed/cisplatin combination – 500 mg/m^2^ and 75 mg/m^2^ Q3W, docetaxel/cisplatin combination – 75 mg/m^2^ and 75 mg/m^2^ Q3W, gemcitabine/cisplatin combination – 1250 mg/m^2^ and 75 mg/m^2^ Q3W. S, simulation; D, experimental data.

We cultured tumor cells derived from five patients with MET-amplified non-small cell lung cancer into *in vitro* patient-derived organoids (PDO) and experimentally assessed the resulting cell viability under different cytotoxic treatments. Based on the *in vitro* testing results, we then created 5 PDO-specific virtual patients by introducing inter-individual variation within certain physiological parameters (tumor growth rate, degree of MET amplification, and drug potency) and calibrating model simulation results against the respective *in vitro* dose-response datasets (Figures 8A,B; Supplementary Figures S6A–E, S7A–E, S8A–E). Then, based on the 5 model-based PDO-informed virtual patients, we predicted the clinical efficacy of the different drugs tested *in vitro* and explored alternative dosing regimens (standard doses and lower doses) based on these drugs in the clinical treatment scenarios. Our model simulations suggested that in a specific patient (patient #4), different doses of the TKI drugs used in the clinical treatment scenario would result in highly different response outcomes. Erlotinib at all doses (full clinical dose to 1/4 dose) can maintain this patient in the strong PR-SD range, while this patient under gefitinib treatment (full clinical dose to 1/4 dose) can only achieve weak PR or even PD (Figure 8C). Regarding the simulation results for patient #1 whose PDOs were tested again chemotherapies (and combinations), our simulation results demonstrated that halving the standard dose of pemetrexed (from 500 mg/m^2^ to 250 mg/m^2^) had a greater impact on therapeutic effect than halving the dose of cisplatin (from 75 mg/m^2^ to 37.5 mg/m^2^) in the pemetrexed/cisplatin combination regimen (Figure 8D). Similar trends were also observed in the simulated docetaxel/cisplatin combination regimen. However, in the gemcitabine/cisplatin combination regimen, the impact of any dose reduction was less pronounced. These results suggested that for patient #1 in clinical practice, dose reduction of cisplatin may be considered when using pemetrexed/cisplatin or docetaxel/cisplatin combination regimen. In the gemcitabine/cisplatin combination regimen, dose reduction of both agents may be considered and still a tumor regression response similar to that of the standard full dose can be expected (Figure 8D). Clinical-level simulations of the other 3 patients were presented in the Supplementary Figures S6F–J, S7F–J, S8F–J.

## Discussion

Targeted therapies against MET remain an active area of investigation in oncology research and drug development with numerous clinical trials of MET inhibitors ongoing for MET-aberrant cancers (Yang et al., 2023; Mazieres et al., 2023a). In this work, we developed a multiscale mechanism-based QSP model that integrated data from cellular, animal and patient levels to facilitate discovery of new therapeutic combinations and optimal treatment regimens for patients. This QSP model framework encompasses the complex signaling network downstream of multiple receptors including MET, EGFR, ALK and ROS1, and it was iterative calibrated and validated against a substantial body of preclinical-clinical data. While the current model focuses primarily on patient populations that were naïve to MET-targeted treatments, it can also be adapted to simulate patients that are already resistant to MET-targeted drugs. Resistance to MET TKIs is now an essential challenge in the clinic and can be caused by common on-target alterations including MET gene amplification and point mutations within the MET kinase domain such as D1228N and G1090A (Botting et al., 2015; Fujino et al., 2019). In addition, resistance can arise through the compensatory activation of alternative receptor-mediated signaling pathways (e.g., EGFR, ALK, RET, ROS1), aberrant stimulation of downstream kinases such as PI3K/AKT/mTOR and RAS/MAPK (Gimenez-Xavier et al., 2017). Research has found that certain type II MET TKIs such as cabozantinib (with MET and other RTKs as targets) can overcome resistance induced by the MET Y1230C mutation that emerged after savolitinib use (Piper-Vallillo et al., 2020). Our model is thus capable of simulating MET TKI resistance driven by diverse mechanisms and offers a unique route to comprehensively evaluate different combination strategies to therapeutically overcome such resistance in the clinic. Still, limitations of the current model should be noted, such as estimation of intratumoral drug distribution and simplified modeling of certain chemotherapeutic drugs’ mechanism of cytotoxicity. Future efforts can further expand on such directions with more arising data.

Using our QSP modeling framework, we demonstrated its potential in terms of simulating population-level efficacy to different therapeutic strategies in both *METex14* mutated and MET-amplified NSCLC patients. Our simulations (Figure 6) predicted that MET TKIs (such as capmatinib, tepotinib) indicated for *METex14* patients can also lead to tumor regression in MET-amplified patients in general, albeit the objective response rates would be lower than in the *METex14* mutated patients. This is consistent with the results reported by the Geometry Mono-1 clinical trial, in which researchers evaluated the efficacy of capmatinib in the aforementioned two patient subpopulations and the observed ORRs were 41% and 36.5% respectively. A number of preclinical research has also shown that MET TKIs are capable of attenuating proliferative signal transduction of cancer cells with normal MET and MET amplification (Tanizaki et al., 2011b; Ramesh et al., 2023). In addition, as MET TKIs are known to induce nontrivial adverse events in patients (e.g., in GEOMETRY mono-1 trial with capmatinib, 67% of patients experienced grade 3 or above AEs, 23% patients had AE-related dose reduction), we explored the model utility in terms of implementing clinical dose optimization. By simulating and comparing the clinical ORRs of different dosing regimens, we proposed that in terms of efficacy, the clinically approved dose for tepotinib, capmatinib and crizotinib was rather optimal, while for savoltinib similar efficacy can be achieved at lower doses, indicating potential space for further dose optimization. It should be noted that with the Project Optimus initiative being more and more crucial throughout the clinical development of new oncology drugs, model-based methodologies like the QSP framework we formulated here can play increasingly important roles in informing accurate clinical design and implementation to ensure optimal dosages of oncology drugs are delivered to patients at bedside (Samineni et al., 2024).

Using the QSP model, we also predicted the potential clinical response of various drug combinations and provided comparative insights for future clinical drug investigations. Although to our knowledge, the model-proposed treatment framework of combining MET TKIs with downstream kinase inhibitors (e.g., MEK, ERK, AKT inhibitors) has not yet entered clinical testing for MET-aberrant lung cancer patients, similar routines (of combining mutation-targeted TKIs with kinase inhibitors) have proven successful in other point mutation-driven NSCLC (e.g., BRAF) (Planchard et al., 2024). Furthermore, two recent studies have tested the combination of MET inhibitor (crizotinib) with MEK inhibitors in late-stage cancer patients (of various cancer types, not selected regarding MET status) and demonstrated preliminary disease-controlling efficacy, which supported the proposed value of MET combinations as predicted by our model simulations (Aroldi et al., 2025; Gallagher et al., 2025). As QSP models of such kind have significant potential in terms of proposing and evaluating new combinations (Wang et al., 2023; Jafarnejad et al., 2019; Zhou et al., 2024), it is also of great interest to incorporate more therapeutics in addition to small molecule inhibitors but relevant for MET-aberrant cancers in the future versions of the model. Currently, novel therapeutic modalities targeting MET-aberrant NSCLC are being actively explored in the clinic. Amivantamab, a bispecific antibody targeting EGFR and MET, was recently approved by the FDA to treat NSCLC patients and it has demonstrated exciting potential in treating MET-aberrant patients according to the CHRYSALIS study (Park et al., 2021). Telisotuzumab Vedotin, an antibody-drug conjugate with MET as the target, was recently approved (based on the LUMINOSITY trial) to treat NSCLC patients with high MET protein overexpression (≥50% of tumor cells with strong/3+ staining) who have received a prior systemic therapy (Camidge et al., 2024). Lately, bispecific antibody drug conjugates targeting EGFR and MET have also attracted attention in the pharmaceutical industry with multiple drugs of this kind already in early phase trials for MET-aberrant cancers (Gong, 2024). By integrating more mechanisms of action of new drug modalities and more therapeutic targets into the current model framework while leveraging more extensive patient-derived organoid testing, we envision that our QSP model framework can be extended to become a comprehensive hypothesis-generating platform. Such QSP platforms can effectively guide clinical candidate selection and combination trial design, as well as provide prospective screening for personalized treatment decision-making in MET-aberrant cancer patients.

## Data Availability

The original contributions presented in the study are included in the article/Supplementary Material, further inquiries can be directed to the corresponding authors.

## Supplementary material

SUPPLEMENTARY TABLE S1 A summary of all model species, reactions, parameters and their descriptions.
