# Supplementary material for: Combining mechanistic quantitative systems pharmacology modeling and patient-derived organoid testing in MET-aberrant non-small cell lung cancer for high-throughput combination efficacy analysis and personalized treatment design
Source: Front Pharmacol. 2025 Nov 24;16:1685468. doi: 10.3389/fphar.2025.1685468 (PMC12682884; doi:10.3389/fphar.2025.1685468)
Supplement: Supplementary file 1 [file DataSheet1.pdf]

Supplementary Materials

Combining Mechanistic Quantitative Systems Pharmacology Modeling and Patient-Derived Organoid Testing in MET-aberrant Non-Small Cell Lung Cancer for High-throughput Combination Efficacy Analysis and Personalized Treatment Design

Supplementary Fig S1. Additional model calibration using pathway signal transduction data and tepotinib treatment data. See also Fig. 2.

Supplementary Fig S2. Additional model calibration using cell viability data treated by various TKIs. See also Fig. 3.

Supplementary Fig S3. QSP submodules describing PK of various drugs in mouse.

Supplementary Fig S4. QSP submodules describing PK of various drugs in human.

Supplementary Fig S5. Additional model calibration using in vivo tumor growth inhibition data in response to chemotherapeutic drugs. See also Fig. 5.

Supplementary Fig S6. Combined PDO-QSP modeling results for patient #2 in terms of clinical efficacy prediction. See also Fig. 8.

Supplementary Fig S7. Combined PDO-QSP modeling results for patient #3 in terms of clinical efficacy prediction. See also Fig. 8.

Supplementary Fig S8. Combined PDO-QSP modeling results for patient #1 and patient #5 in terms of clinical efficacy prediction. See also Fig. 8.

Supplementary Fig S9. Detailed diagram of the QSP model structure. See also Fig. 1.

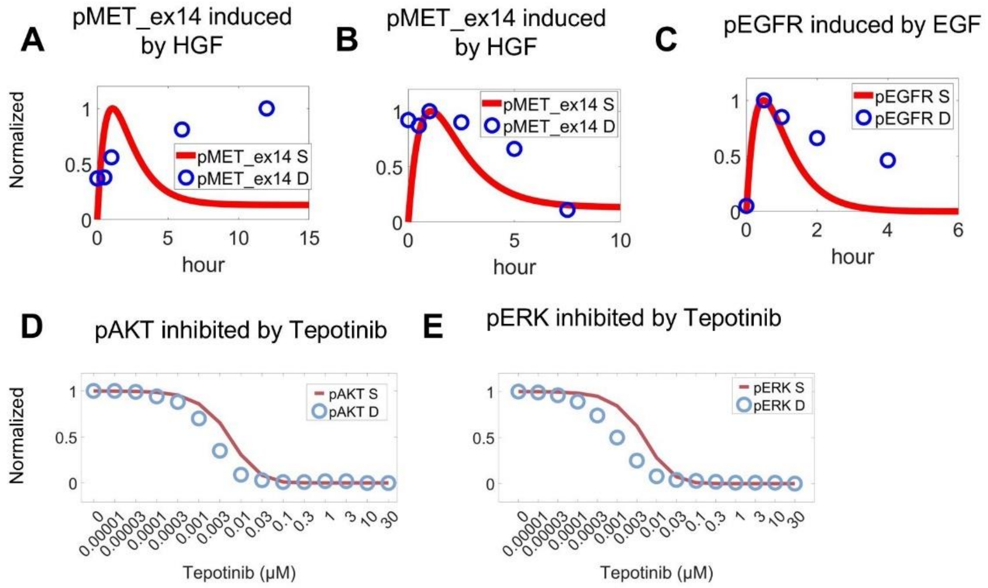

Figure S1. Additional model calibration using pathway signal transduction data and tepotinib treatment data. The cell-level QSP model accurately reproduced the following additional experimental data including: (**A**, **B**) HGF (50ng/ml, 100 ng/mL) induces phosphorylation of MET receptors with exon 14 skipping mutations [1, 2], (**C)** EGF (100 ng/mL) induces time-dependent phosphorylation of EGFR [3], **(D, E)** Phosphorylation of AKT and ERK can be inhibited by tepotinib in a dose-dependent manner [4]. Y axes are relative expression levels (normalized to their respective maximum values). S, simulation; D, experimental in vitro data.

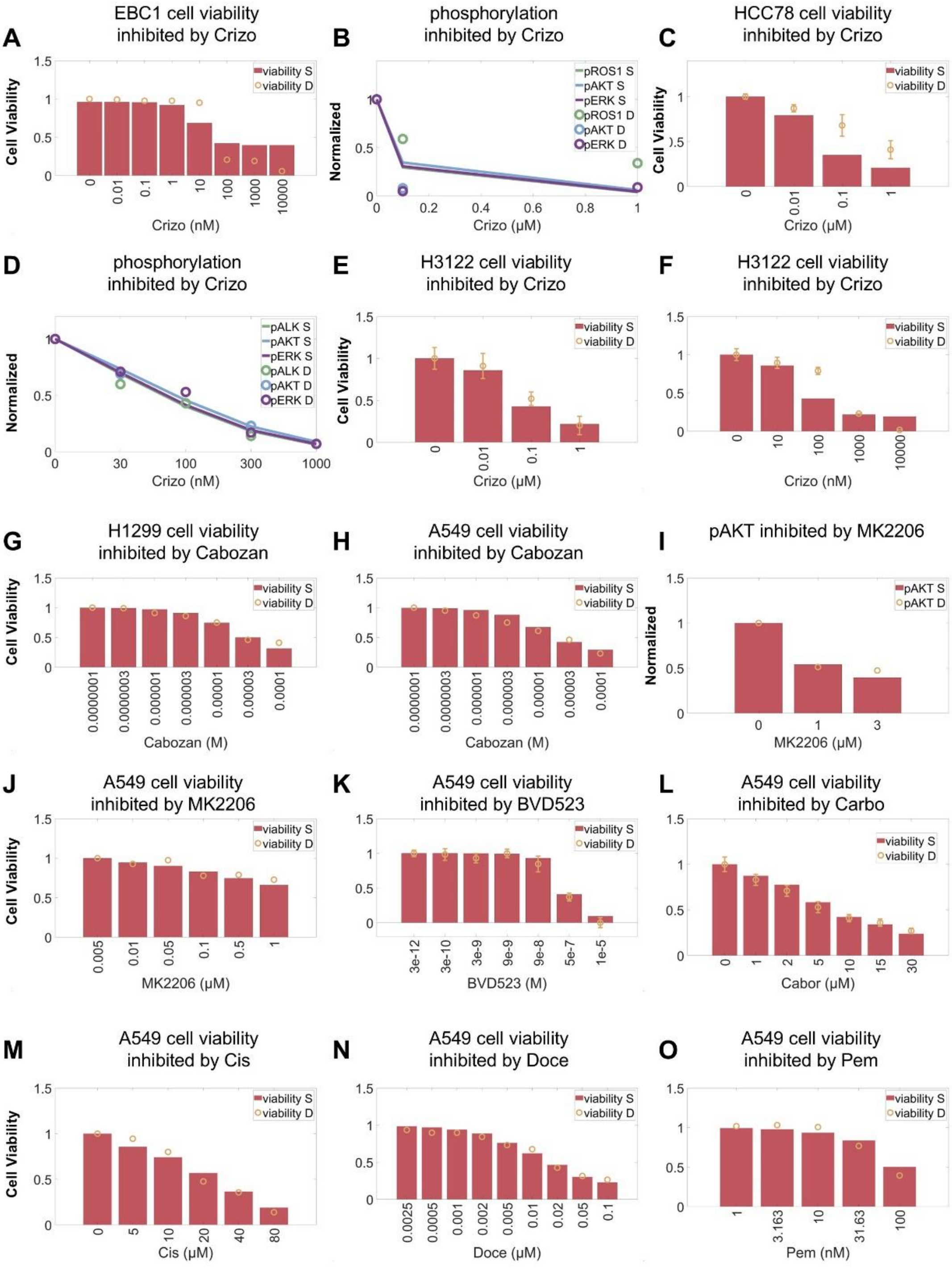

Figure S2. Additional model calibration using cell viability data treated by various TKIs. The cell-level QSP model accurately reproduced the following additional experimental data including: **(A)** dose-dependent inhibition of cell viability by crizotinib [5], **(B, C)** inhibition of

pAKT, pERK and pROS1 by crizotinib in ROS1-rearranged HCC78 cells [6], **(D-F)** inhibition of pAKT, pERK, pALK and cell viability by crizotinib in ALK-mutant H3122 cells [6, 7], **(G, H)** inhibition of cell viability by cabozantinib in MET-normal NSCLC cell lines H1299 and A549 [8], **(I, J)** inhibition of pAKT and cell viability by MK2206 (AKT inhibitor) [9], **(K)** inhibition of cell viability by BVD523 (ERK inhibitor) [10], **(L-O)** dose-dependent inhibition of cell viability by chemotherapeutic agents (carboplatin, cisplatin, docetaxel, and pemetrexed) [11-15]. Y axes are relative expression levels (normalized to their respective maximum values). S, simulation; D, experimental in vitro data.

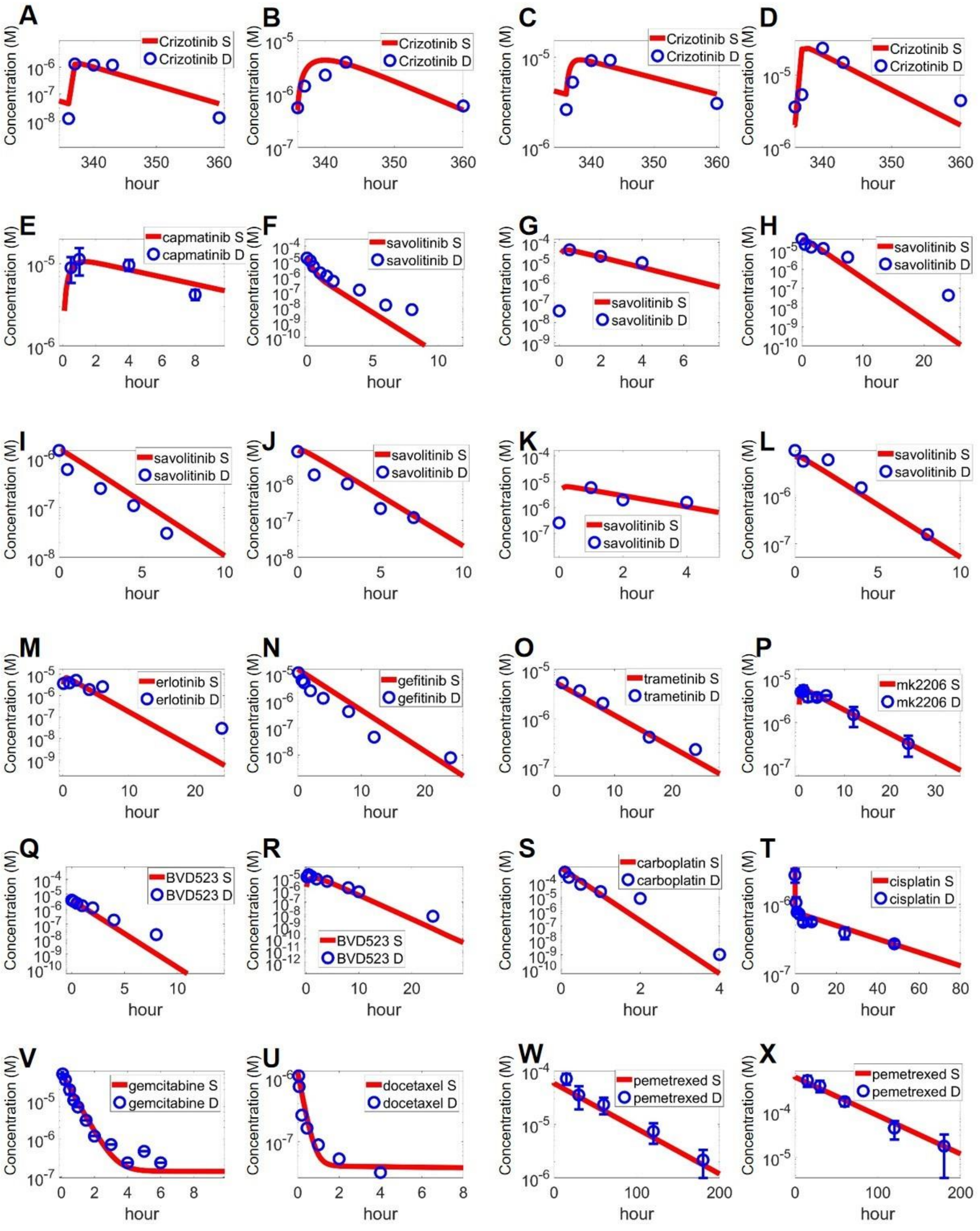

Figure S3. QSP submodules describing PK of various drugs in mouse. In the QSP model, we incorporated PK submodules of various drugs in mouse that are calibrated against published data, including: **(A-D)** plasma concentrations of crizotinib at various doses (25, 50, 100, and 200 mg/kg, QD, oral administration) on day 14 post-administration [16]; **(E)** plasma concentrations of capmatinib following a single oral administration of 25 mg/kg [17]; **(F)** plasma concentrations of savolitinib following a single intravenous injection of 2.5 mg/kg [18], **(G-H)** plasma concentrations of savolitinib following oral administration of 100 mg/kg QD and 100mg/kg Q2D [18], **(I)** plasma concentrations of savolitinib following oral administration of 12.5 mg/kg QD [18], **(J)** plasma concentrations of savolitinib following oral

administration of 25 mg/kg QD [18], **(K-L)** plasma concentrations of savolitinib following oral administration of 30 mg/kg BID or QD [18]; **(M)** plasma concentrations of erlotinib following a single oral dose of 10 mg/kg [19]; **(N)** plasma concentrations of gefitinib following a single intravenous injection of 20 mg/kg [20]; **(O)** plasma concentrations of trametinib following a single intravenous injection of 5 mg/kg [21]; **(P)** plasma concentrations of MK2206 following a single oral dose of 120 mg/kg [22]; **(Q)** plasma concentrations of BVD523 following a single intravenous injection of 1 mg/kg, **(R)** plasma concentrations of BVD523 following a single oral administration of 10 mg/kg [23]; **(S)** plasma concentrations of carboplatin following a single intravenous injection of 60 mg/kg [24]; **(T)** plasma concentrations of cisplatin following a single intravenous injection of 1.25 mg/kg [25]; **(U)** plasma concentrations of gemcitabine following a single intravenous injection of 100 mg/kg [26]; **(V)** plasma concentrations of docetaxel following a single intravenous injection of 76 nmol/g [27]; **(W, X)** plasma concentrations of pemetrexed following a single intravenous injection of 10 and 100 mg/kg [28]. S, simulation; D, published PK data in mouse.

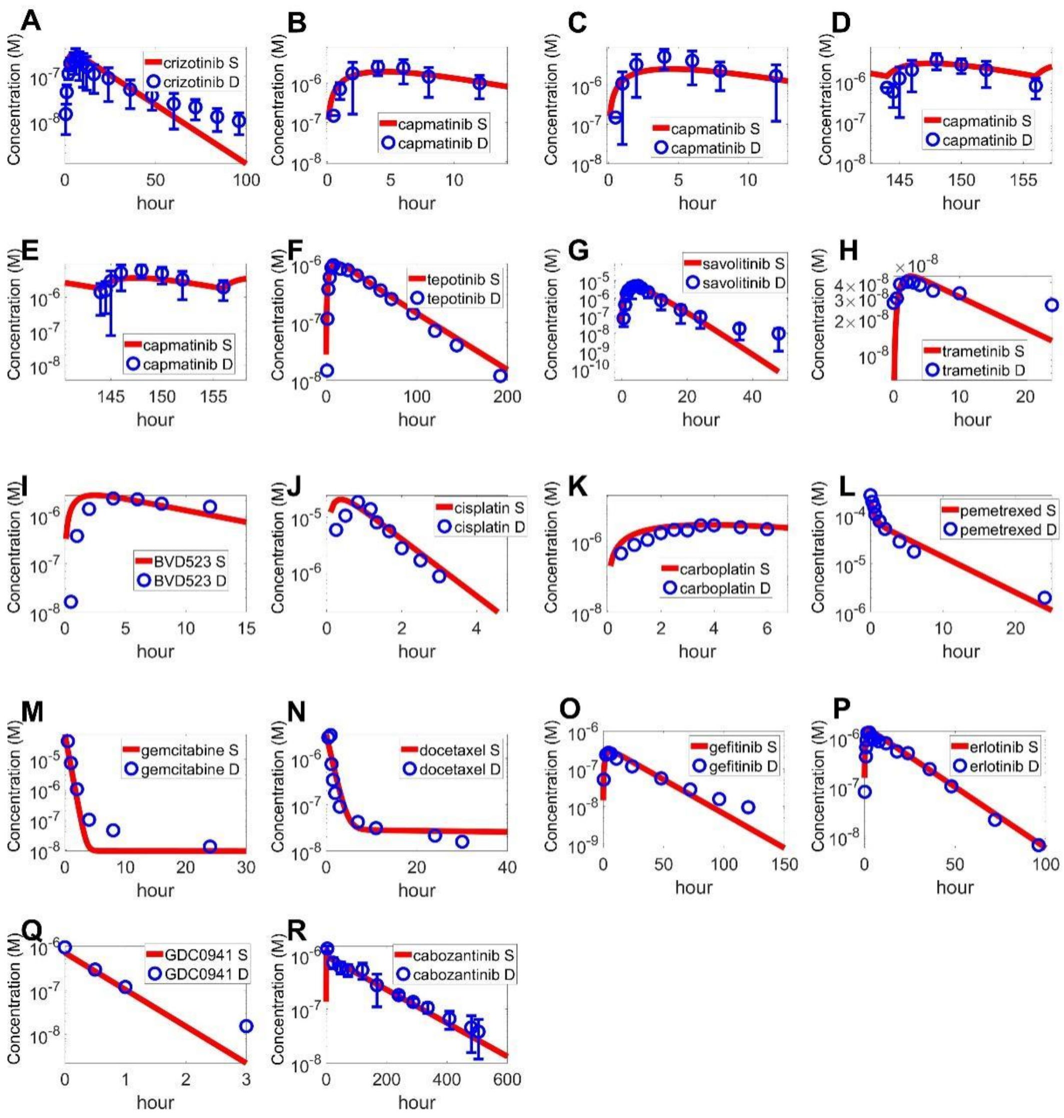

Figure S4. QSP submodules describing PK of various drugs in human. In the QSP model, the aforementioned PK submodules of various drugs in mouse were scaled to predict human PK and are calibrated against published data, including:: **(A)** crizotinib (250 mg, single oral dose) [29]; **(B, C)** capmatinib (300 mg/kg BID and 400 mg/kg BID, oral administration, day 1); **(D, E)** capmatinib (300 mg/kg BID and 400 mg/kg BID, oral administration, day 7) [30]; **(F)** tepotinib (500 mg, single oral dose); **(G)** savolitinib (600 mg, single oral dose) [31]; **(H)** trametinib (2 mg, single oral dose); (I) BVD523 (600 mg, single oral dose) [32]; **(J)** cisplatin (100 mg/m², single subcutaneous injection); **(K)** carboplatin (100 mg/m², single subcutaneous injection) [33]; **(L)** pemetrexed (500 mg/m², single intravenous injection) [34]; **(M)** gemcitabine (1000 mg/m², single intravenous injection); **(N)** docetaxel (100 mg/m², single intravenous injection) [35]; **(O)** gefitinib (250 mg, single oral dose); **(P)** erlotinib (100 mg, single oral dose) [36]; **(Q)** GDC0941 (1 mg, single intravenous injection) [37]; **(R)** cabozantinib (140 mg, single intravenous injection) [38]. S, simulation; D, published PK data in human.

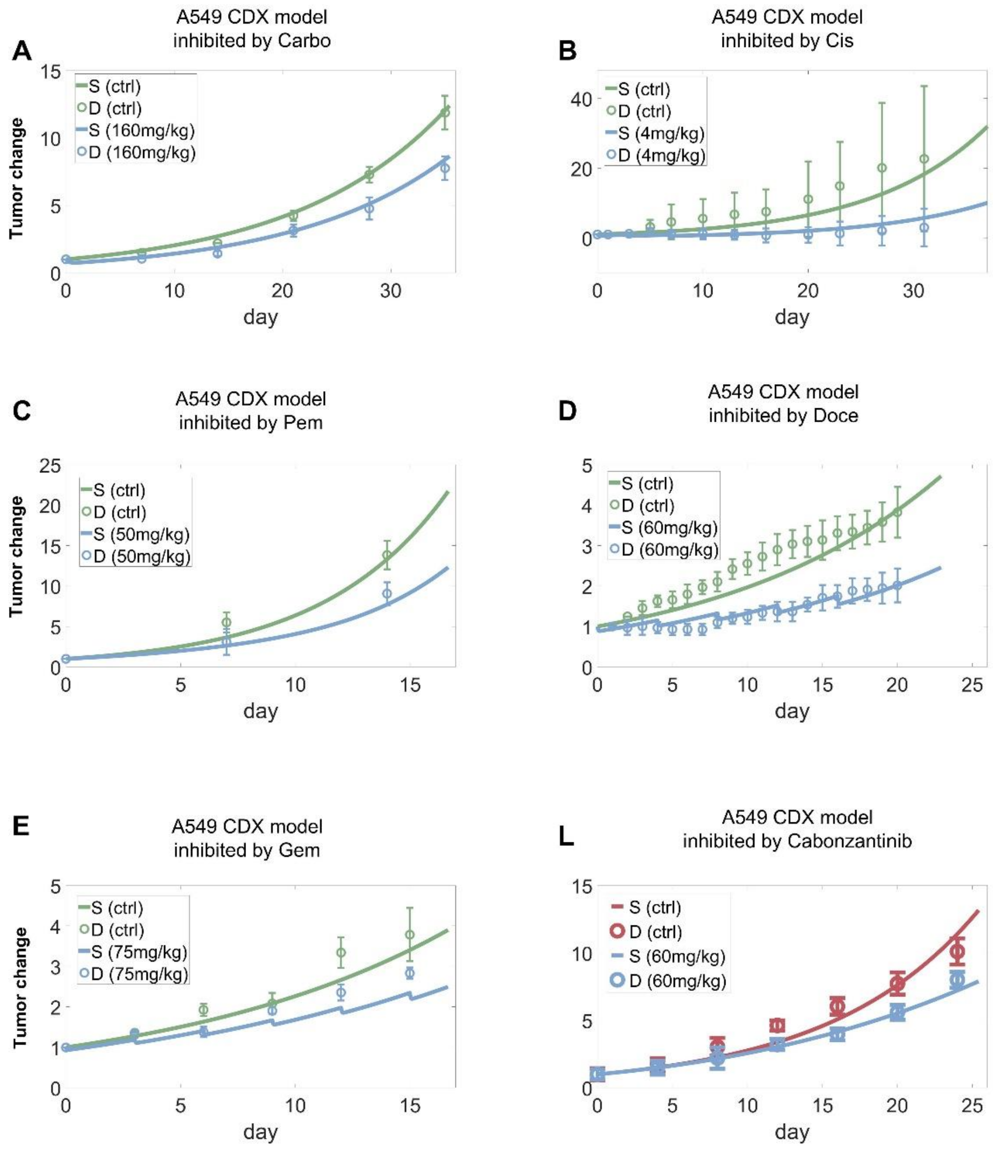

Figure S5. Additional model calibration using in vivo tumor growth inhibition data in response to chemotherapeutic drugs. Additional QSP model calibration using the following in vivo time-course tumor growth inhibition data: **(A)** carboplatin (160 mg/kg) [24]; **(B)** cisplatin (4 mg/kg) [39]; **(C)** pemetrexed (50 mg/kg) [40]; **(D)** docetaxel (60 mg/kg) [41]; **(E)** gemcitabine (75 mg/kg) [42] ; **(E)** cabozantinib (60 mg/kg) [43]. All tumor growth profiles were normalized to their starting volumes. The weight of a mouse was assumed to be approximately 20 g to calculate the drug doses administered. S, simulation; D, experimental *in vivo* data**.**

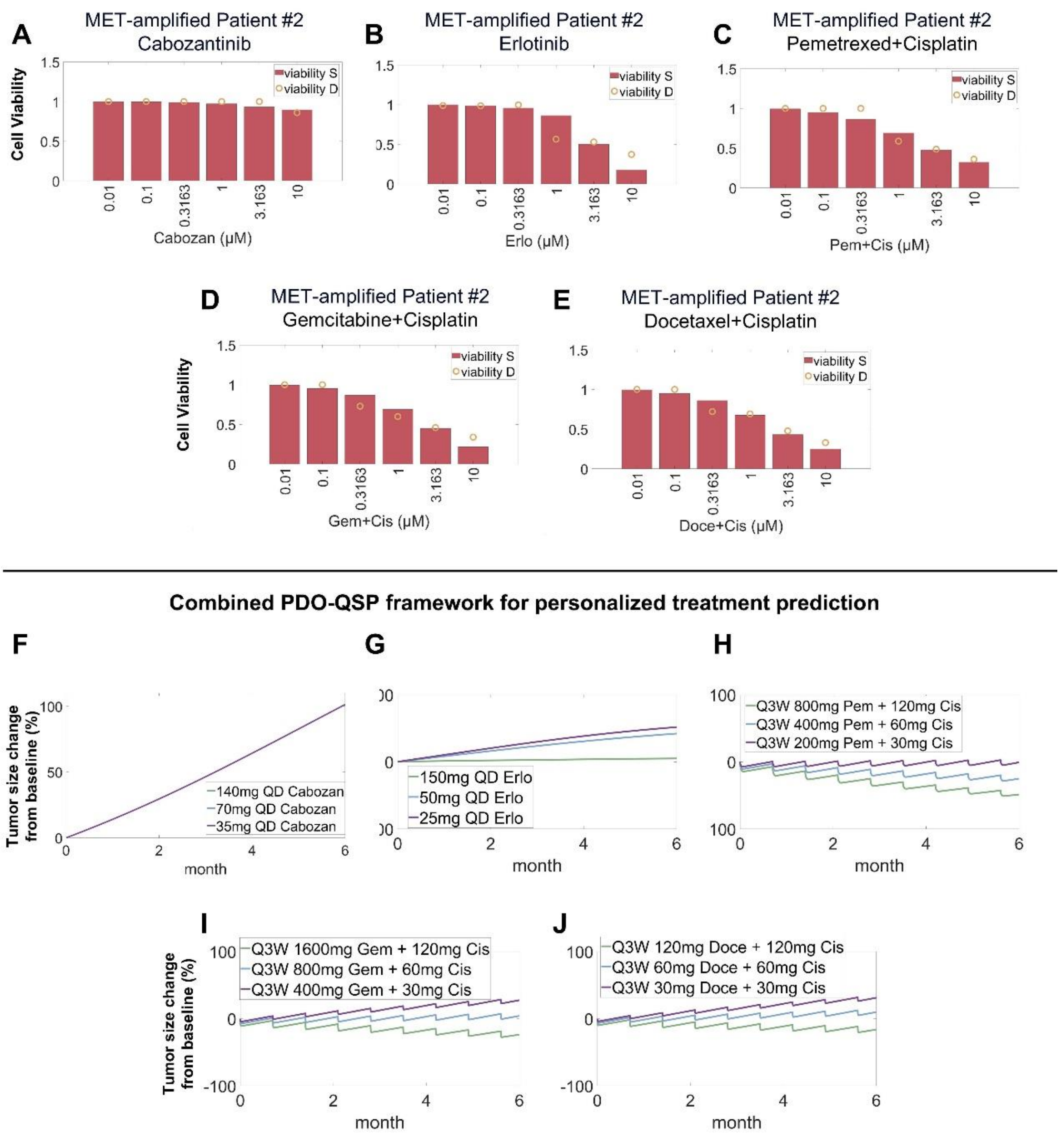

Figure S6. Combined PDO-QSP modeling results for patient #2 in terms of clinical efficacy prediction. (A-E) Patient-derived organoids (showing patient #2 here) from MET-amplified NSCLC patients were cultured in vitro and treated with different candidate drugs (cabozantinib, erlotinib, pemetrexed combined with cisplatin, gemcitabine combined with cisplatin, docetaxel combined with cisplatin) at different doses, and experimental cell viability results were used to inform QSP model parameterization during the formation of this individualized virtual patient. **(F-J)** The combined PDO-QSP model framework was then utilized to predict and compare the individual patient's clinical tumor regression profiles under different potential dosing regimens. Full drug dose/schedule used in simulations: cabozantinib – 140 mg QD, erlotinib – 150 mg QD, pemetrexed/cisplatin combination – 500 mg/m² and 75 mg/m² Q3W, docetaxel/cisplatin combination – 75 mg/m² and 75 mg/m² Q3W, gemcitabine/cisplatin combination – 1250 mg/m² and 75 mg/m² Q3W. S, simulation; D, experimental data**.**

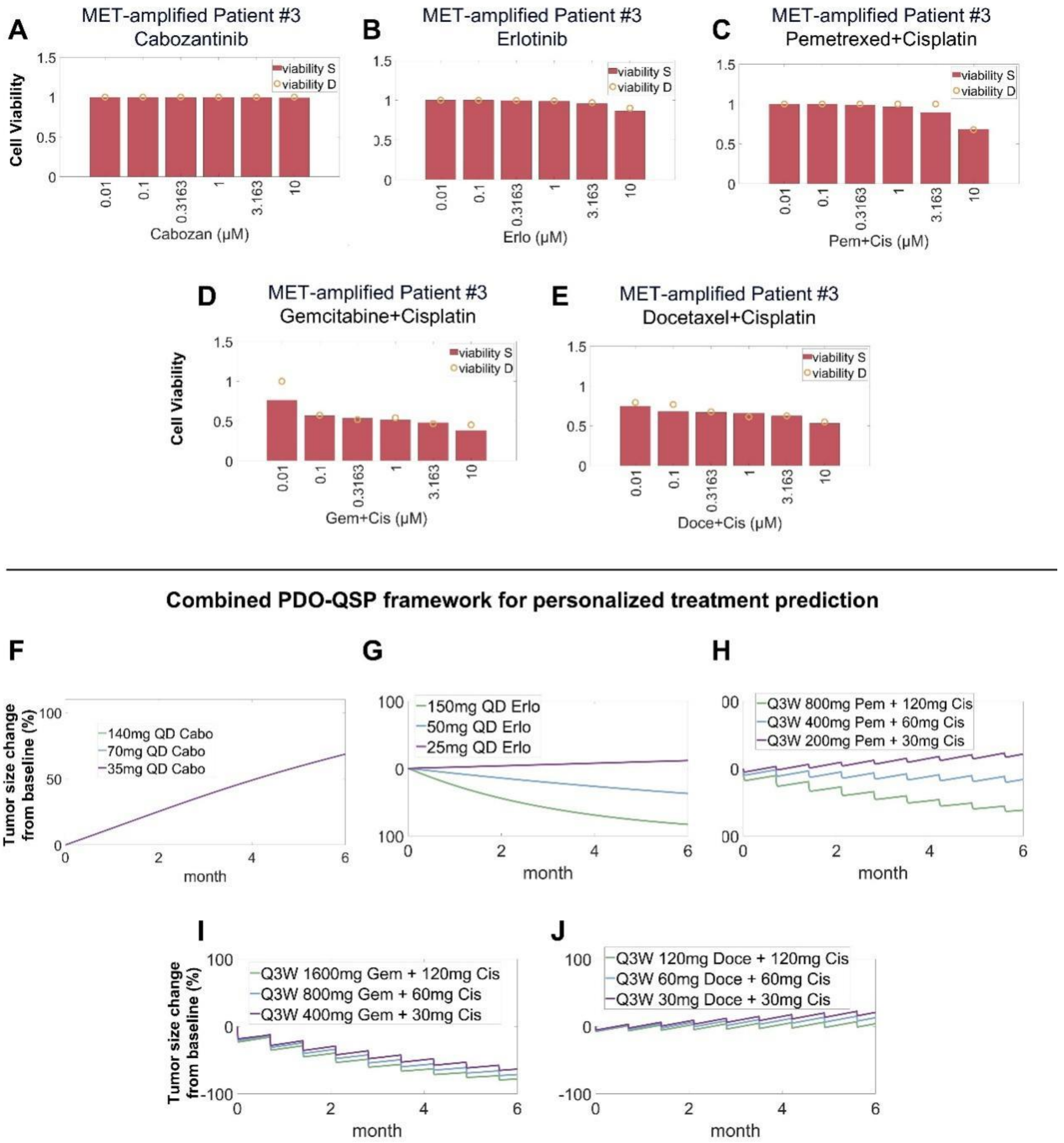

Figure S7. Combined PDO-QSP modeling results for patient #3 in terms of clinical efficacy prediction. (A-E) Patient-derived organoids (showing patient #3 here) from MET-amplified NSCLC patients were cultured in vitro and treated with different candidate drugs (cabozantinib, erlotinib, pemetrexed combined with cisplatin, gemcitabine combined with cisplatin, docetaxel combined with cisplatin) at different doses, and experimental cell viability results were used to inform QSP model parameterization during the formation of this individualized virtual patient. **(F-J)** The combined PDO-QSP model framework was then utilized to predict and compare the individual patient's clinical tumor regression profiles under different potential dosing regimens. Full drug dose/schedule used in simulations: cabozantinib – 140 mg QD, erlotinib – 150 mg QD, pemetrexed/cisplatin combination – 500 mg/m² and 75 mg/m² Q3W, docetaxel/cisplatin combination – 75 mg/m² and 75 mg/m² Q3W, gemcitabine/cisplatin combination – 1250 mg/m² and 75 mg/m² Q3W. S, simulation; D, experimental data**.**

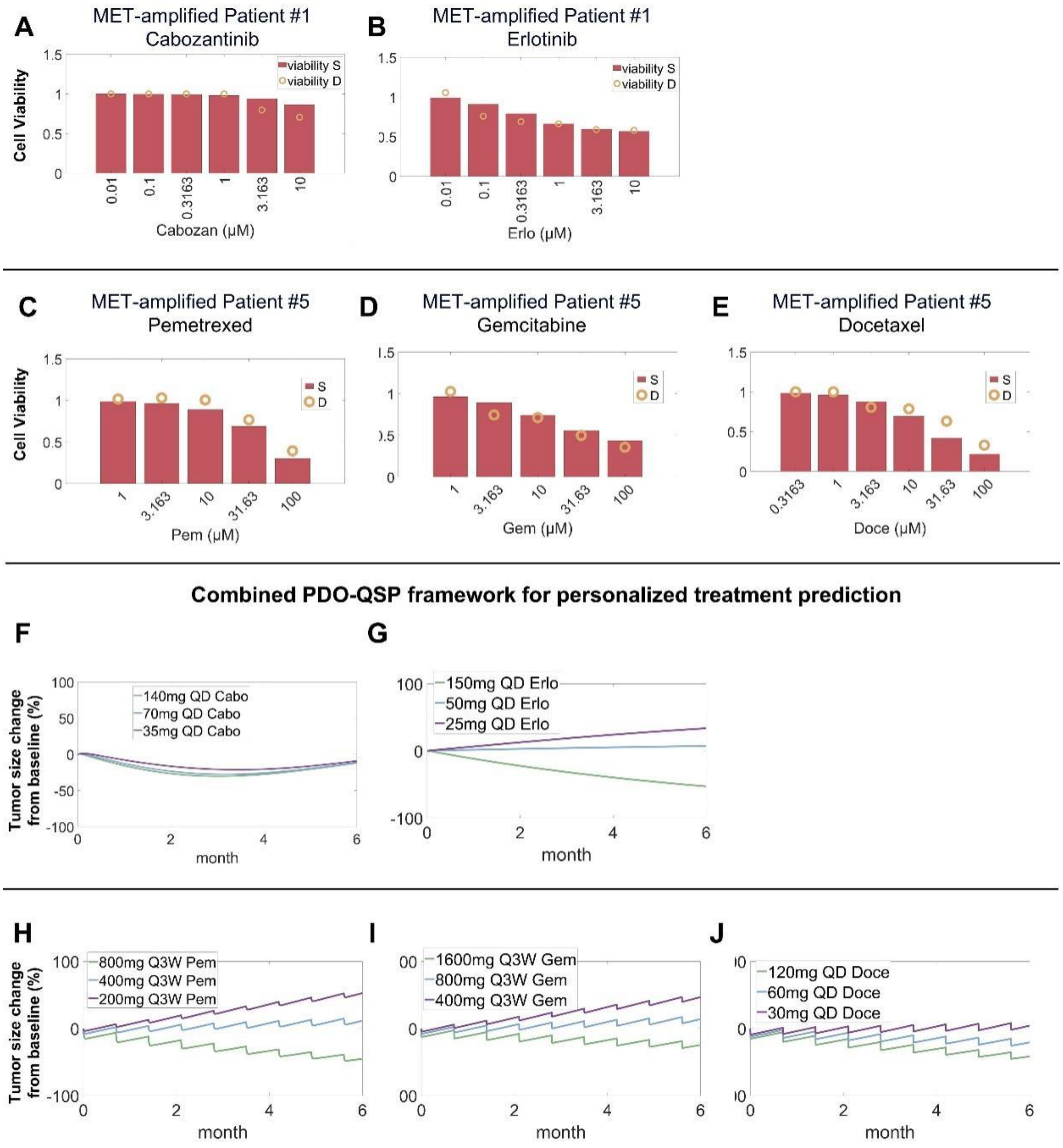

Figure S8. Combined PDO-QSP modeling results for patient #1 and patient #5 in terms of clinical efficacy prediction. (A-E) Patient-derived organoids (showing patient #1 and #5 here) from MET-amplified NSCLC patients were cultured in vitro and treated with different candidate drugs (cabozantinib, erlotinib, pemetrexed, gemcitabine, docetaxel) at different doses, and experimental cell viability results were used to inform QSP model parameterization during the formation of this individualized virtual patient. **(F-J)** The combined PDO-QSP model framework was then utilized to predict and compare the individual patient's clinical tumor regression profiles under different potential dosing regimens. Full drug dose/schedule used in simulations: cabozantinib – 140 mg QD, erlotinib – 150 mg QD, pemetrexed – 500 mg/m² Q3W, docetaxel – 75 mg/m² Q3W, gemcitabine – 1250 mg/m² Q3W. S, simulation; D, experimental data**.**

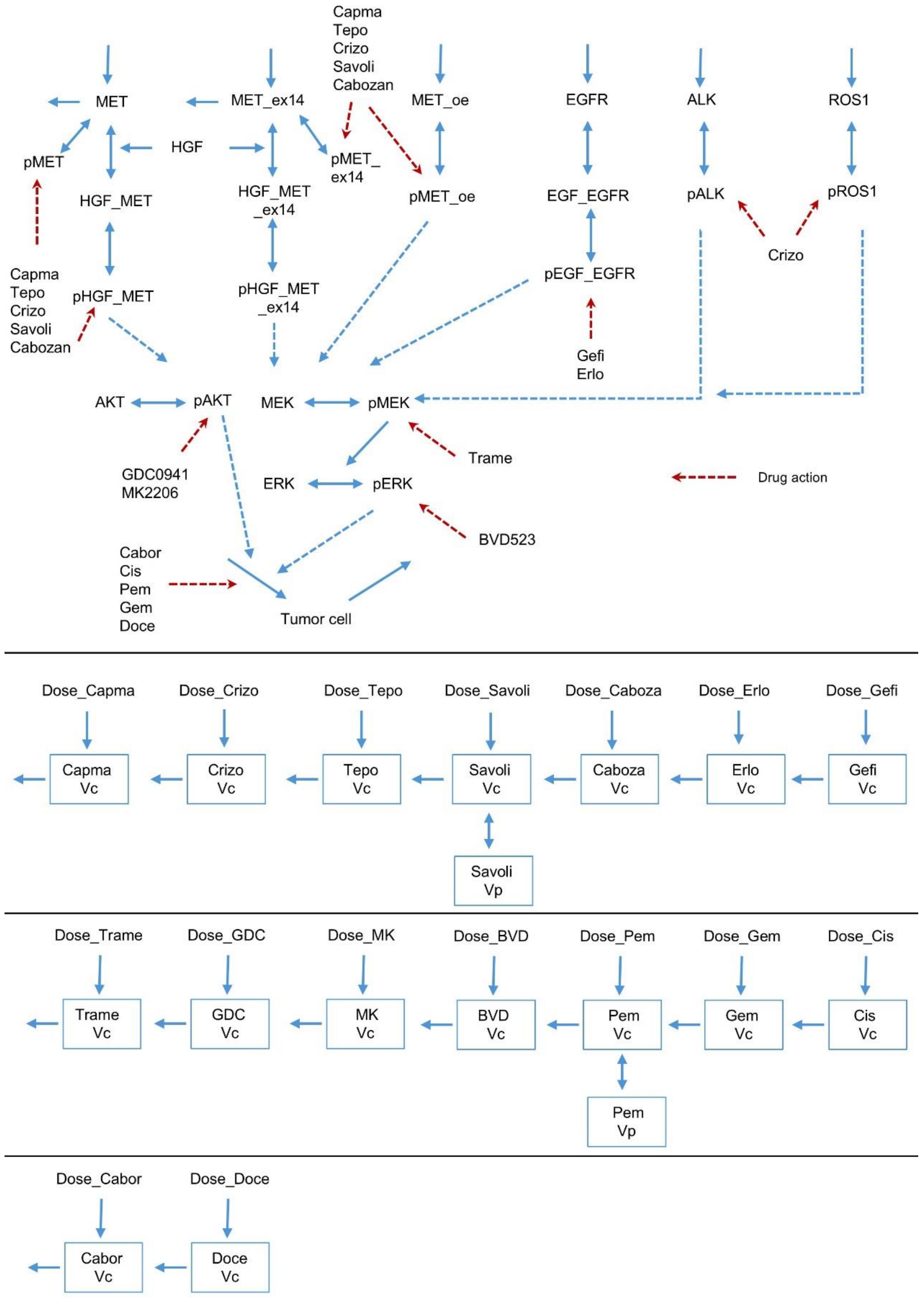

Figure S9. Detailed diagram of the QSP model structure. All unique model species and reaction fluxes were shown with labels. See more details in Supplemental Tables S1.

References

1. Ahn, H.K., et al., *MET Receptor Tyrosine Kinase Regulates the Expression of Co-Stimulatory and Co-Inhibitory Molecules in Tumor Cells and Contributes to PD-L1-Mediated Suppression of Immune Cell Function.* Int J Mol Sci, 2019. **20**(17).
2. Wang, F., et al., *Functional Analysis of MET Exon 14 Skipping Alteration in Cancer Invasion and Metastatic Dissemination.* Cancer Res, 2022. **82**(7): p. 1365-1379.
3. Nishimura, Y., et al., *EGF-stimulated AKT activation is mediated by EGFR recycling via an early endocytic pathway in a gefitinib-resistant human lung cancer cell line.* Int J Oncol, 2015. **46**(4): p. 1721-9.
4. Albers, J., et al., *The Preclinical Pharmacology of Tepotinib-A Highly Selective MET Inhibitor with Activity in Tumors Harboring MET Alterations.* Mol Cancer Ther, 2023. **22**(7): p. 833-843.
5. Kim, S., et al., *Acquired Resistance of MET-Amplified Non-small Cell Lung Cancer Cells to the MET Inhibitor Capmatinib.* Cancer Res Treat, 2019. **51**(3): p. 951-962.
6. Yasuda, H., et al., *Preclinical rationale for use of the clinically available multitargeted tyrosine kinase inhibitor crizotinib in ROS1-translocated lung cancer.* J Thorac Oncol, 2012. **7**(7): p. 1086-90.
7. Jorge, S.E., et al., *Responses to the multitargeted MET/ALK/ROS1 inhibitor crizotinib and co-occurring mutations in lung adenocarcinomas with MET amplification or MET exon 14 skipping mutation.* Lung Cancer, 2015. **90**(3): p. 369-74.
8. Karmacharya, U., et al., *Novel Pyridine Bioisostere of Cabozantinib as a Potent c-Met Kinase Inhibitor: Synthesis and Anti-Tumor Activity against Hepatocellular Carcinoma.* Int J Mol Sci, 2021. **22**(18).
9. Dai, B., et al., *KEAP1-dependent synthetic lethality induced by AKT and TXNRD1 inhibitors in lung cancer.* Cancer Res, 2013. **73**(17): p. 5532-43.
10. Shin, M., C.E. Franks, and K.L. Hsu, *Isoform-selective activity-based profiling of ERK signaling.* Chem Sci, 2018. **9**(9): p. 2419-2431.
11. Shi, S., et al., *ER stress and autophagy are involved in the apoptosis induced by cisplatin in human lung cancer cells.* Oncol Rep, 2016. **35**(5): p. 2606-14.
12. Wu, H.M., et al., *Gemcitabine-Induced Autophagy Protects Human Lung Cancer Cells from Apoptotic Death.* Lung, 2016. **194**(6): p. 959-966.
13. Wang, Y., et al., *Downregulation of breast cancer resistance protein by long-term fractionated radiotherapy sensitizes lung adenocarcinoma to SN-38.* Invest New Drugs, 2021. **39**(2): p. 458-468.
14. Liu, Y., C. He, and X. Huang, *Metformin partially reverses the carboplatin-resistance in NSCLC by inhibiting glucose metabolism.* Oncotarget, 2017. **8**(43): p. 75206-75216.
15. Kwon, J.H., et al., *Afatinib Overcomes Pemetrexed-Acquired Resistance in Non-Small Cell Lung Cancer Cells Harboring an EML4-ALK Rearrangement.* Cells, 2019. **8**(12).

16. Yamazaki, S., et al., *Pharmacokinetic/pharmacodynamic modeling of crizotinib for anaplastic lymphoma kinase inhibition and antitumor efficacy in human tumor xenograft mouse models.* J Pharmacol Exp Ther, 2012. **340**(3): p. 549-57.
17. Zuckermann, M., et al., *Capmatinib is an effective treatment for MET-fusion driven pediatric high-grade glioma and synergizes with radiotherapy.* Mol Cancer, 2024. **23**(1): p. 123.
18. Jones, R.D.O., et al., *A pharmacokinetic-pharmacodynamic model for the MET tyrosine kinase inhibitor, savolitinib, to explore target inhibition requirements for anti-tumour activity.* Br J Pharmacol, 2021. **178**(3): p. 600-613.
19. Smith, N.F., et al., *Modulation of erlotinib pharmacokinetics in mice by a novel cytochrome P450 3A4 inhibitor, BAS 100.* Br J Cancer, 2008. **98**(10): p. 1630-2.
20. Bi, Y., et al., *A Whole-Body Physiologically Based Pharmacokinetic Model of Gefitinib in Mice and Scale-Up to Humans.* Aaps j, 2016. **18**(1): p. 228-38.
21. Vaidhyanathan, S., et al., *Factors influencing the CNS distribution of a novel MEK-1/2 inhibitor: implications for combination therapy for melanoma brain metastases.* Drug Metab Dispos, 2014. **42**(8): p. 1292-300.
22. Chang, L.S., et al., *Brigatinib causes tumor shrinkage in both NF2-deficient meningioma and schwannoma through inhibition of multiple tyrosine kinases but not ALK.* PLoS One, 2021. **16**(7): p. e0252048.
23. Suresh, P.S., et al., *Prediction of Human Pharmacokinetics of Ulixertinib, a Novel ERK1/2 Inhibitor from Mice, Rats, and Dogs Pharmacokinetics.* Eur J Drug Metab Pharmacokinet, 2018. **43**(4): p. 453-460.
24. Wang, H., et al., *Pretreatment with dexamethasone increases antitumor activity of carboplatin and gemcitabine in mice bearing human cancer xenografts: in vivo activity, pharmacokinetics, and clinical implications for cancer chemotherapy.* Clin Cancer Res, 2004. **10**(5): p. 1633-44.
25. Levet, V., et al., *Platinum pharmacokinetics in mice following inhalation of cisplatin dry powders with different release and lung retention properties.* Int J Pharm, 2017. **517**(1-2): p. 359-372.
26. Thompson, B.R., et al., *Pharmacokinetics of gemcitabine and its amino acid ester prodrug following intravenous and oral administrations in mice.* Biochem Pharmacol, 2020. **180**: p. 114127.
27. de Graan, A.J., et al., *Influence of polymorphic OATP1B-type carriers on the disposition of docetaxel.* Clin Cancer Res, 2012. **18**(16): p. 4433-40.
28. Greillier, L., et al., *Intrapleural administration of pemetrexed: a pharmacokinetic study in an animal model.* J Thorac Oncol, 2009. **4**(3): p. 404-8.
29. Johnson, T.R., et al., *Metabolism, excretion and pharmacokinetics of [14C]crizotinib following oral administration to healthy subjects.* Xenobiotica, 2015. **45**(1): p. 45-59.
30. Glaenzel, U., et al., *Absorption, Distribution, Metabolism, and Excretion of Capmatinib (INC280) in Healthy Male Volunteers and In Vitro Aldehyde Oxidase Phenotyping of the Major Metabolite.* Drug Metab Dispos, 2020. **48**(10): p. 873-885.
31. Xiong, W., et al., *Population pharmacokinetic analysis of tepotinib, an oral MET*

kinase inhibitor, including data from the VISION study. Cancer Chemother Pharmacol, 2022. **89**(5): p. 655-669.
32. Voon, P.J., et al., *Phase I pharmacokinetic study of single agent trametinib in patients with advanced cancer and hepatic dysfunction.* J Exp Clin Cancer Res, 2022. **41**(1): p. 51.
33. Andersson, A., et al., *Pharmacokinetics of cisplatin and its monohydrated complex in humans.* J Pharm Sci, 1996. **85**(8): p. 824-7.
34. Kavathiya, K., et al., *A Comparative Pharmacokinetic Study of 2 Pemetrexed Formulations in Indian Adult Chemonaive Patients With Adenocarcinoma Stage III/IV Non-Small Cell Lung Cancer.* Clin Pharmacol Drug Dev, 2017. **6**(3): p. 234-239.
35. Derissen, E.J.B., et al., *Intracellular pharmacokinetics of gemcitabine, its deaminated metabolite 2',2'-difluorodeoxyuridine and their nucleotides.* Br J Clin Pharmacol, 2018. **84**(6): p. 1279-1289.
36. Bergman, E., et al., *Pharmacokinetics of gefitinib in humans: the influence of gastrointestinal factors.* Int J Pharm, 2007. **341**(1-2): p. 134-42.
37. Salphati, L., et al., *Preclinical pharmacokinetics of the novel PI3K inhibitor GDC-0941 and prediction of its pharmacokinetics and efficacy in human.* Xenobiotica, 2011. **41**(12): p. 1088-99.
38. Nguyen, L., et al., *Pharmacokinetics of cabozantinib tablet and capsule formulations in healthy adults.* Anticancer Drugs, 2016. **27**(7): p. 669-78.
39. Zhou, D., et al., *Overcoming tumor resistance to cisplatin through micelle-mediated combination chemotherapy.* Biomater Sci, 2015. **3**(1): p. 182-91.
40. Booth, L., et al., *PDE5 inhibitors enhance the lethality of pemetrexed through inhibition of multiple chaperone proteins and via the actions of cyclic GMP and nitric oxide.* Oncotarget, 2017. **8**(1): p. 1449-1468.
41. Feng, S.Q., et al., *Combined treatment with apatinib and docetaxel in A549 xenograft mice and its cellular pharmacokinetic basis.* Acta Pharmacol Sin, 2018. **39**(10): p. 1670-1680.
42. Wang, R., et al., *Efficacy of inverso isomer of CendR peptide on tumor tissue penetration.* Acta Pharm Sin B, 2018. **8**(5): p. 825-832.
43. Alhazzani, K., et al., *Augmented antitumor effects of erlotinib and cabozantinib on A549 non-small cell lung cancer: In vitro and in vivo studies.* Saudi Pharm J, 2023. **31**(10): p. 101756.
